# Comparison of Manual and Automated Preprocedural Segmentation Tools to Predict the Annulus Plane Angulation and C-Arm Positioning for Transcatheter Aortic Valve Replacement

**DOI:** 10.1371/journal.pone.0151918

**Published:** 2016-04-13

**Authors:** Verena Veulemans, Tobias Zeus, Laura Kleinebrecht, Jan Balzer, Katharina Hellhammer, Amin Polzin, Patrick Horn, Alexander Blehm, Jan-Philipp Minol, Patric Kröpil, Ralf Westenfeld, Tienush Rassaf, Artur Lichtenberg, Malte Kelm

**Affiliations:** 1 Department of Medicine, Division of Cardiology, Pulmonary Diseases, Vascular Medicine, University Hospital Düsseldorf, Düsseldorf, Germany; 2 Clinic for Cardiovascular Surgery, Medical Faculty, University Hospital Düsseldorf, Düsseldorf, Germany; 3 Institute of Radiology, University Hospital Düsseldorf, Düsseldorf, Germany; University of Messina, ITALY

## Abstract

**Background:**

Preprocedural manual multi-slice-CT-segmentation tools (MSCT-ST) define the gold standard for planning transcatheter aortic valve replacement (TAVR). They are able to predict the perpendicular line of the aortic annulus (PPL) and to indicate the corresponding C-arm angulation (CAA). Fully automated planning-tools and their clinical relevance have not been systematically evaluated in a real world setting so far.

**Methods and Results:**

The study population consists of an all-comers cohort of 160 consecutive TAVR patients with a drop out of 35 patients for technical and anatomical reasons. 125 TAVR patients underwent preprocedural analysis by manual (M-MSCT) and fully automated MSCT-ST (A-MSCT). Method-comparison was performed for 105 patients (Cohort A). In Cohort A, CAA was defined for each patient, and accordance within 10° between M-MSCT and A-MSCT was considered adequate for concept-proof (95% in LAO/RAO; 94% in CRAN/CAUD). Intraprocedural CAA was defined by repetitive angiograms without utilizing the preprocedural measurements. In Cohort B, intraprocedural CAA was established with the use of A-MSCT (20 patients). Using preprocedural A-MSCT to indicate the corresponding CAA, the levels of contrast medium (ml) and radiation exposure (cine runs) were reduced in Cohort B compared to Cohort A significantly (23.3±10.3 vs. 35.3 ±21.1 ml, p = 0.02; 1.6±0.7 vs. 2.4±1.4 cine runs; p = 0.02) and trends towards more safety in valve-positioning could be demonstrated.

**Conclusions:**

A-MSCT-analysis provides precise preprocedural information on CAA for optimal visualization of the aortic annulus compared to the M-MSCT gold standard. Intraprocedural application of this information during TAVR significantly reduces the levels of contrast and radiation exposure.

**Trial Registration:**

ClinicalTrials.gov NCT01805739

## Introduction

Transcatheter aortic valve replacement (TAVR) is a percutaneous procedure for patients with severe and symptomatic aortic stenosis who are unable to undergo surgical aortic valve replacement or who have an increased operative risk [1–3]. Crucial for the success of this minimally invasive procedure is an in depth understanding of the aortic root anatomy. The exact measurement of the aortic annulus provides information about the diameter, circumference, area, and correct perpendicular line of the aortic annulus (PPL) for choosing an adequate corresponding C-arm angulation (CAA) and the correct prosthesis size [4–6].

Choosing the best “implanter’s view” with the use of various fluoroscopic projections during the TAVR procedure provides a safe implantation strategy, but it depends on repetitive angiograms in different CAAs, requiring multiple injections of contrast medium and a prolonged radiation time. Preprocedural evaluation of the PPL may help to optimize the implantation process by predicting the perfect intraprocedural orientation. There are several software tools that facilitate the preprocedural planning of PPL.

The accuracy of a fully automated preprocedural planning and guidance tool for predicting the PPL has not previously been systematically compared to manual MSCT-segmentation software. The aim of the present study was to I) evaluate annulus plane angulation and CAA obtained by a fully automated segmentation tool in comparison to manual standard (method comparison) and II) analyze the clinical benefit in patients undergoing TAVR when preprocedural CAA-data are implemented to define the intraprocedural PPL compared to the standard “follow the right cusp” approach or delivery-adapted orientation forms.

## Methods

### Study Population

All consecutive patients with severe aortic stenosis who received TAVR at the Heart Center Duesseldorf between March 2014 and January 2015 were potentially eligible for inclusion. The study population consisted of 160 patients who underwent TAVR with either the CoreValve system (Medtronic Inc., Minneapolis, MN), Engager system (Medtronic Inc., Minneapolis, MN) or Edwards SAPIEN 3 Valve (Edwards Lifesciences, Irvine, CA). All patients gave written informed consent for TAVR and the use of clinical, procedural and follow up data for research. The study procedures were in accordance with the Declaration of Helsinki, and the institutional Ethics Committee of the Heinrich-Heine University approved the study protocol (4080). The study is registered at clinical trials (NCT01805739). Preprocedural MSCT was routinely performed in all patients before TAVR. For automated MSCT analysis (A-MSCT), HeartNavigator^®^ (Philips, Eindhoven, Netherlands) and for manual MSCT-analysis (M-MSCT), OsiriX MD (64 bit, FDA cleared, CE II labeled, for clinical use) was used.

In the first 105 patients, method-comparison was performed (Cohort A). The intraprocedural CAA was established without utilization of the preprocedural measurements. Preprocedural CAA was defined for each patient, and accordance within 10 degrees was considered adequate. Intraprocedural PPL was identified by the “follow the right cusp”-technique with sequential aortic root angiographies (Edwards Sapien valve, Engager valve) or by a combination of aortic root angiographies and strict perpendicular view of the distal radiopaque ring of the delivery system (Corevalve).

**Fig 1:**
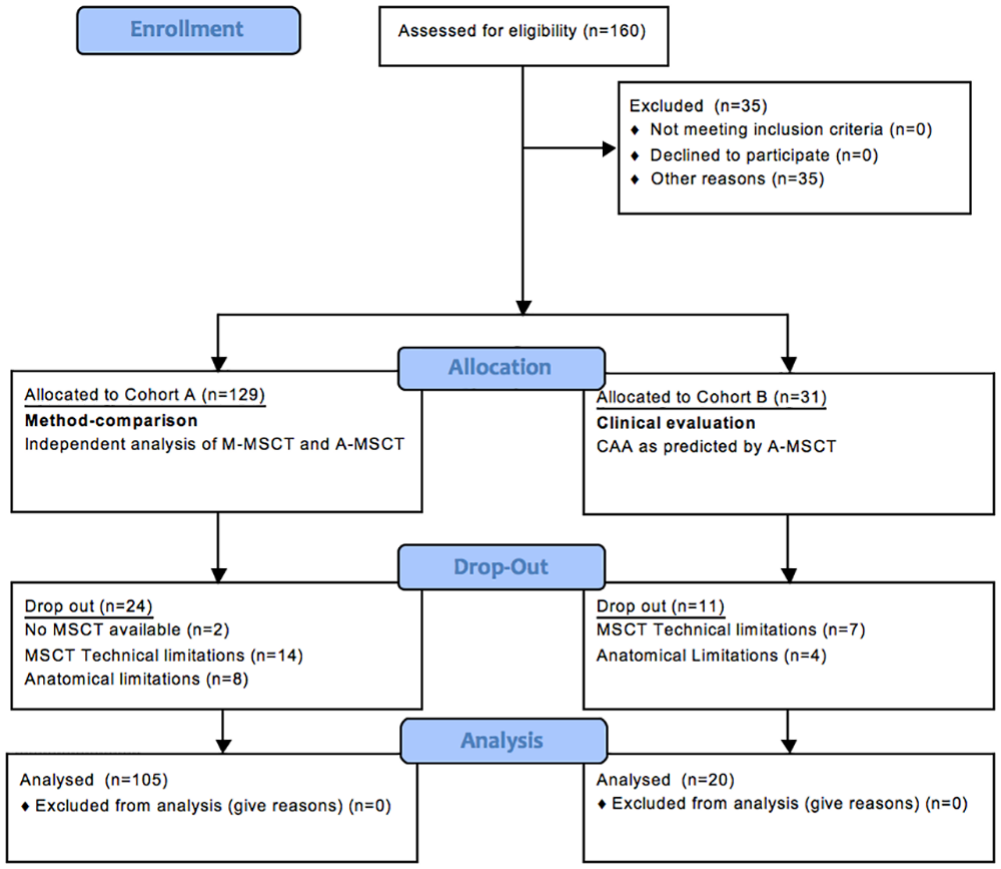
Flowchart of the Study. The study population consists of an all-comers cohort of 160 consecutive TAVR patients and 35 patients have been excluded. **Cohort A:** CT-datasets in this cohort were analyzed to perform method-comparison (see text, n = 105). Automated MSCT analysis software (A-MSCT) and manual software (M-MSCT) were used to determine the perpendicular valve plane angulation (PPL). **Cohort B:** Here, intraprocedural C-arm angulation (CAA) was established with the use of A-MSCT.

In Cohort B (20 patients), the intraprocedural CAA was established with the use of A-MSCT. CAA-correspondence was analyzed and levels of contrast medium and radiation exposure were compared to Cohort A. From a total of 160 TAVR-patients, 35 were excluded from CT analysis because of technical or anatomical limitations (no available MSCT, motion artefacts, slice thickness>1 mm, valve-in-valve procedures, bicuspid aortic valve, e.g.) (Fig 1).

### MSCT Image Acquisition Protocol

CT data were obtained using a 128-slice, single source CT-scanner (“SOMATOM Definition AS+”, Siemens Healthcare, Forchheim, Germany) with a high temporal resolution of 150 ms and a collimation of 128×0.6 mm according to TAVR-related standardized recommendations for CT image acquisition [5]. All examinations were ECG-gated and were performed using automated tube potential selection and tube current modulation. The pitch was 0.2. After a timing bolus scan, contrast material (75 ml Iomeprol 400 mg/ml, Imeron 400 MCT^®^, Bracco Imaging, Milan, Italy) was injected, and followed by saline solution (flow rate of 4 ml/s). Axial images were reconstructed in the best diastolic phase with a slice thickness of 0.6 mm at a reconstruction increment of 0.3. Additional multiplanar reformation (MPR) of the aortic root and annular plane were achieved with a width of ≤1 mm to achieve high spatial resolution according to TAVR-related standardized recommendations for CT image acquisition.

### Automated 3D Image Analysis of MSCT

**Fig 2:**
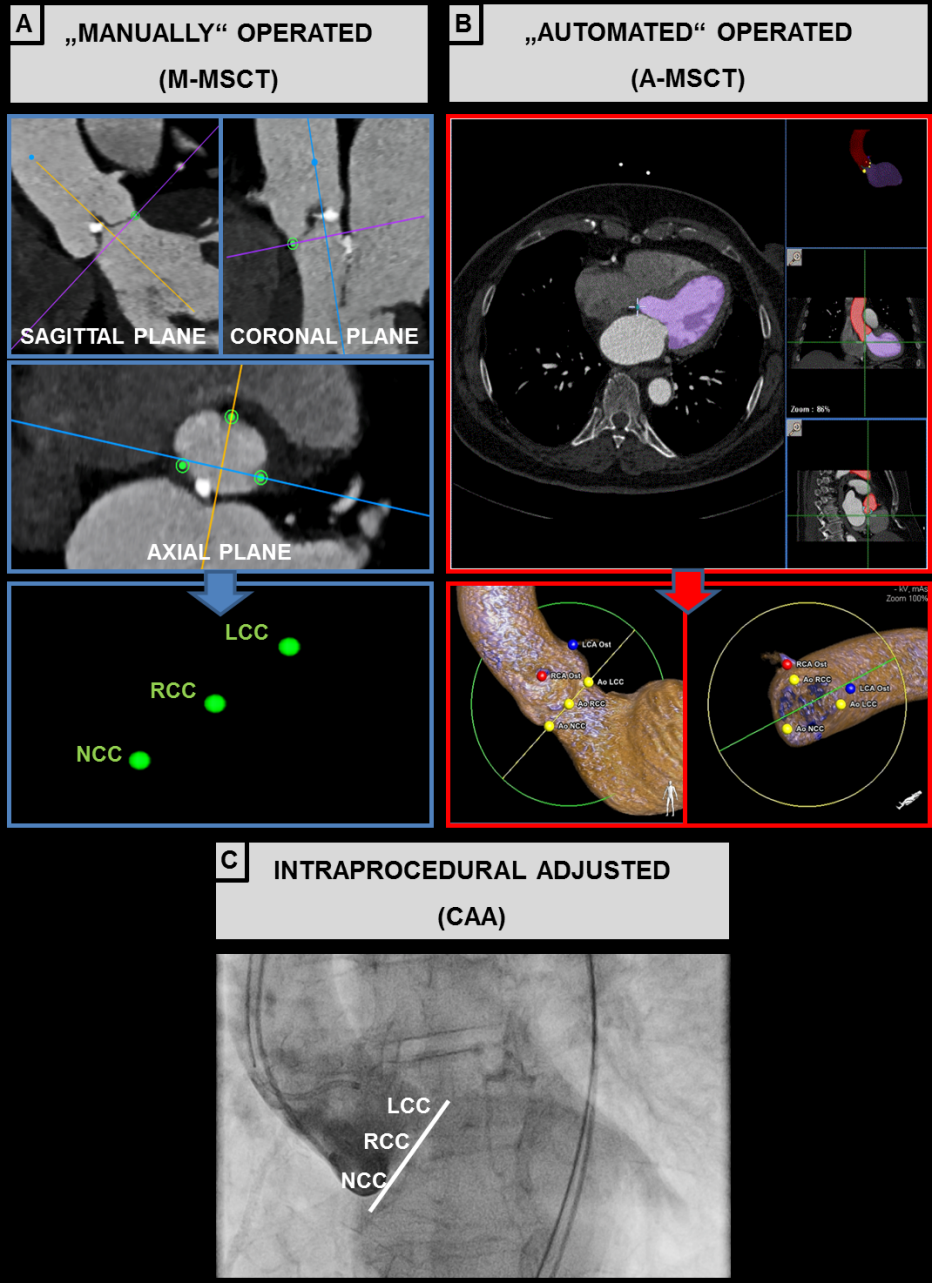
Preprocedural Alignment of the Aortic Root Planes. Colored lines through selected CT images reflect the 3D schematic reconstructions in several planes using a manual software (M-MSCT) **(A, coronal, sagittal and axial planes)**. The axial plane presents the basis for the alignment of the hinge point plane, in which no valve structure is visible (hinge points). Three points were set on the axial plane, and the 3D volume-rendered reconstruction was initiated. The angles were determined by manually rotating the 3D aortic reconstructions to reach the appropriate projection with a perpendicular view. The automated software (A-MSCT) automatically places fiducial marks at the hinge points (yellow points), representing the aortic valve plane **(B)**. The aortic root angiogram displays a perpendicular valve view on the aortic valve annulus **(C)**. NCC = noncoronary cusp; RCC = right coronary cusp; LCC = left coronary cusp; LAO = left anterior oblique; CAUD = caudal; CRAN = cran; M-MSCT = Manual derived CAA by MSCT; A-MSCT = Automated derived CAA by MSCT; CAA = Intraprocedural C-arm angulation.

A-MSCT was performed by a commercially available interventional planning and guidance tool, intended to simplify the planning, measurement, device selection and projection angle selection in preparation for the TAVR procedure. By integrating CT datasets, the heart is automatically segmented to visualize anatomical structures and landmarks and provides insights into the calcification distribution in the ascending aorta, aortic valve and left ventricle. The software automatically places fiducial marks in a circle of 4 mm, at the hinge points of the three deep touching aortic valve leaflets, representing the aortic valve plane [7]. Optimal key CAA, defined by its combination of cranio-caudal (CRAN-CAUD) and right anterior oblique / left anterior oblique (RAO-LAO) angulation, was adjusted into a “3-bulbi implanter’s view”, whereby the C-arm is perpendicular to the annulus plane and the RCC is located centrally between the NCC at the right and the LCC at the left. The adjusted PPL was read after visual analysis of the correct segmentation procedure for all necessary landmarks (Fig 2).

### Manual 3D Image Analysis of MSCT

M-MSCT was performed by a multimodality post-processing advanced open-source PACS workstation with clinical applications. It was designed for the display and interpretation of large sets of multidimensional and multimodality images, and it offers all modern rendering modes [8]. For quantitative analysis, three reference planes intersecting at 90° to one another were defined. To determine the predicted PPL, MSCT-datasets were reconstructed in the coronal, sagittal and axial planes with the use of workstation tools. After placement in the accurate annular valve plane, three points were set on the axial plane, and 3-dimensional (3D) volume-rendered reconstruction was initiated. Angles were determined by manually rotating 3D aortic reconstructions to reach the appropriate projection with a perpendicular view (Fig 2).

### TAVR Procedure, Angiographic Image Acquisition and Analysis

The TAVR procedures were performed according to the current guidelines [9]. Transfemoral aortic valve replacement (TF AVR) was performed under local anesthesia. Transapical aortic valve replacement (TA AVR) was performed under general anesthesia. Both procedures were performed in the hybrid suite at the Heart Center Düsseldorf. For Cohort A, the preprocedural analyses with M-MSCT and A-MSCT were performed independent from each other by 2 different and highly experienced operators. The intraprocedural deployment projection was adjusted according to the “following the right cusp”-strategy or according to closure of the distal radiopaque ring of the valve delivery system. Intraprocedural CAA was established by repetitive aortic root angiograms with injection of 15 ml of diluted radiographic contrast through a 5F pigtail catheter each.

In contrast, for Cohort B the preprocedural A-MSCT derived, recommended CAA was used for the first aortic root angiogram. If the perpendicular view was not achieved, repetitive angiograms followed.

### Preprocedural Comparison of M-MSCT and A-MSCT for Prediction of Intraprocedural Annular Valve Angulation

**Fig 3:**
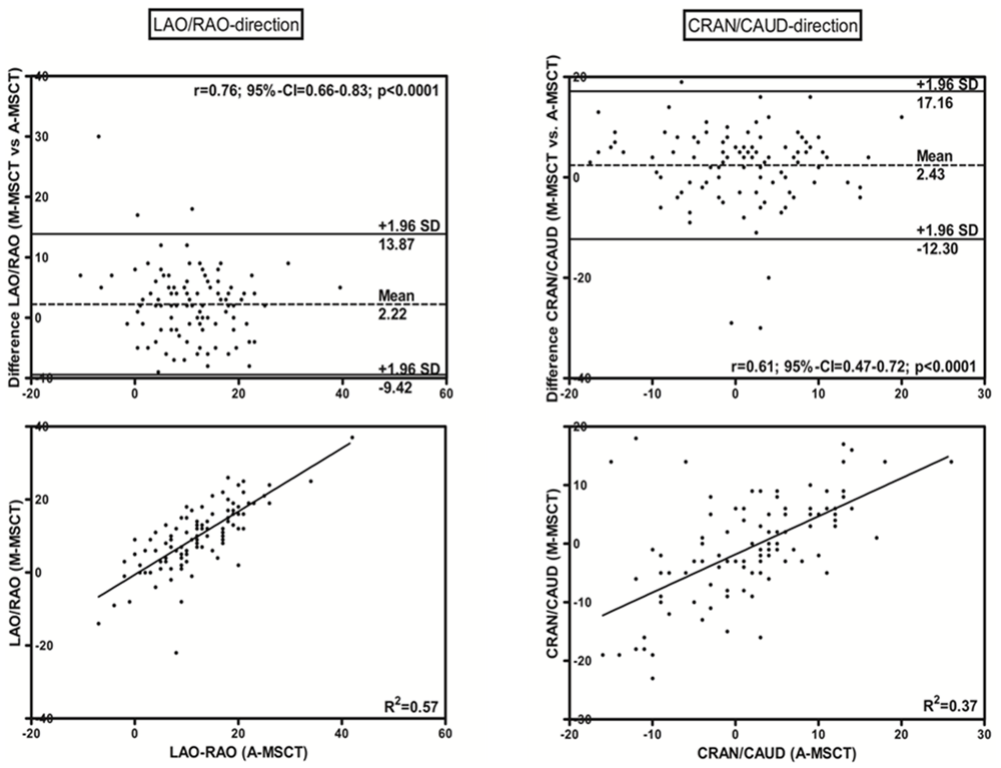
Relationship of the MSCT-derived Prediction of the Perpendicular View Angulation between Automated (A-MSCT) and Manual (M-MSCT) Software (Cohort A, method-comparison). Bland–Altman plots and linear regression analyses comparing M-MSCT and A-MSCT in the LAO/RAO and CRAN/CAUD directions.

The mean PPL was calculated in LAO/RAO- and CRAN/CAUD-(direction) for all available modalities (Table 1). In Cohort A, the mean deviations of the PPL in the were calculated,comparing manual (M-MSCT) to automated (A-MSCT) MSCT-analysis. Accordance within 10 degrees (Δ10°, Table 2) in all directions when comparing the modalities was considered adequate to guarantee accordance. The comparison of A-MSCT and M-MSCT for predicting the PPL and their accordance with the intraprocedural chosen CAA is displayed in the Bland–Altman difference plot with a mean bias and 1.96 standard deviation intervals, as well as by the linear regression model with the Pearson correlation coefficient (Fig 3). LAO and cranial angulation (CRAN) are meant to be positive (+) and RAO and caudal (CAUD) direction are indicated as negative (-) in every figure and calculation.

**Table 1:** Comparison of the Mean Perpendicular Valve Angulations.

(Cohort), Angulation	M-MSCT (Mean)	A-MSCT(Mean)	CAA (Mean)
(A, n = 105) LAO/RAO (°)	+9.8±8.9^a^	+12.2±7.8^a^	+5.0±11.9^a^
(A, n = 105) CRAN/CAUD (°)	-0.7±8.8^b^	+1.8±8.2^b^	-4.8±10.4^b^
(B, n = 20) LAO/RAO (°)	-	+13.0±9.6^c^	+12.3±9.3^c^
(B; n = 20) CRAN/CAUD (°)	-	+1.3±8.6^d^	-2.8±6.7^d^

Values are mean ± SD; Mean perpendicular valve angulations were calculated in LAO/RAO- and CRAN/CAUD-direction for all available modalities. For Cohort A, over-all p-value is calculated by ANOVA Friedman test comparing differencies between all modalities:

^**a)**^p<0.0001

^**b)**^p<0.0001.

For Cohort B, Mann-Whitney t-test was used to compare preprocedural calculated with intraprocedural chosen CAA:

^**c)**^p = 0.8709

^**d)**^p = 0.1474.

M-MSCT = Manual derived CAA by MSCT; A-MSCT = Automated derived CAA by MSCT; CAA = Intraprocedural C-arm angulation; LAO and cranial angulation (CRAN) is meant to be positive **(+)**, RAO and caudal (CAUD) direction is signed to be negative **(-)**; LAO = left anterior oblique; RAO = right anterior.

**Table 2:** Comparison of the Mean Deviation of the Perpendicular Valve Angulations and Correspondance in Cohort A.

Deviation of Angulation	M-MSCT vs. A-MSCT (Mean)	M-MSCT vs. A-MSCT (Accordance within 10°)
**LAO/RAO (°)**	4.9±3.5	100 (95)
**CRAN/CAUD (°)**	5.1±3.6	99 (94)

Values are mean ± SD or n (%); Mean deviation of perpendicular valve angulations were calculated comparing M-MSCT against A-MSCT analysis for Cohort A. Accordance within 10° was meant to be adequate and is shown in number and frequencies. M-MSCT = Manual derived CAA by MSCT; A-MSCT = Automated derived CAA by MSCT; CAA = Intraprocedural C-arm angulation; LAO = left anterior oblique; RAO = right anterior oblique; CAUD = caudal; CRAN

### Data Processing and Statistical Analysis

The collected data included patient characteristics, imaging findings, periprocedural in-hospital data, laboratory results and follow up data. Clinical endpoints were reported according to The Valve Academic Research Consortium (VARC-2) consensus statement [10]. Continuous data are expressed as the mean and standard deviation and were compared using Student’s t-test, Mann-Whitney test and Friedman ANOVA as appropiate. Categorical variables were described by frequencies and percentages. The relationship between the predicted perpendicular valve view projections through several modalities was analyzed using a Bland–Altman difference plot and Pearson correlation method. Pearson correlation coefficients of 0.8 to 1.0 and 0.5 to 0.8, respectively, indicate a very strong and strong positive correlation between two variables, whereas coefficients between 0.2 to 0.5 and 0.0 to 0.2 suggest medium and small correlations, respectively. Data analysis was performed using the statistical software SPSS Statistics 22 (IBM^®^) and GraphPad (Prism^®^). All statistical tests were 2-tailed, and a value of p<0.05 was considered statistically significant. Sample size calculation of cohort B was conducted using G*Power software (Version 3.1; Kiel, Germany). Assuming an alpha error of 0.05, a power of 80% and an effect size of 0.8, 21 patients were needed to detect relevant clinical differences.

## Results

### Baseline Characteristics

125 of 160 patients were included in total, 105 patients in Cohort A and 20 patients in Cohort B. Except for gender distribution, no significant difference in characteristics was observed between the both cohorts. For further details, please see supplementary material (S1 Table).

### Patient Procedural Characteristics and Outcomes

For details on the procedural data and patients’ outcomes, please see Table 3. TAVR was performed by either the transfemoral approach using the CoreValve prosthesis and SAPIEN 3 prosthesis or the transapical approach with SAPIEN prosthesis or Engager prostheses. After thirty days intrahospital death have been documented in 3 cases (3%) in Cohort A and severe postprocedural aortic regurgitation (AR>2) appeared in 2 patients (2%) in Cohort A. In one case post-dilatation was performed to reduce paravalvular regurgitation. In two cases a second prosthesis was implanted (valve-in-valve, 2%). In Cohort B, using preprocedural calculated CAA for best deployment projection, no moderate to severe aortic regurgitation or valve malposition was documented and no post-dilatation or valve-in-valve procedure was necessary.

**Table 3:** Patient Procedural Characteristics and Outcomes.

**A Patient Procedural Characteristics**			
**Procedural Data**	**Cohort A (n = 105)**	**Cohort B (n = 20)**	**p-Value**
TF access, n (%)	82 (78)	16 (80)	>0.9999
TA access, n (%)	23 (22)	4 (20)	>0.9999
EDWARDS, n (%)			
SAPIEN 23 mm, n (%)	7 (7)	4 (20)	0.7503
SAPIEN 26 mm, n (%)	21 (20)	1 (5)	
SAPIEN 29 mm, n (%)	8 (8)	0 (0)	
MEDTRONIC, n (%)			
CoreValve 23 mm, n (%)	1 (1)	0 (0)	0.2480
CoreValve 26 mm, n (%)	18 (17)	4 (20)	
CoreValve 29 mm, n (%)	31 (30)	7 (35)	
CoreValve 31 mm, n (%)	18 (17)	3 (15)	
Engager 23 mm, n (%)	1 (1)	0 (0)	
Engager 26 mm, n (%)	0 (0)	1 (5)	
Contrast administration (ml) ± SD (total)	145.1 ± 78.6	112.5 ± 31.1	0.1567
Contrast agent use until TAVR (ml) ± SD	35.3 ± 21.1	23.3 ± 10.3	0.02
Radiation time (min) ± SD (total)	22.9 ± 30.5	20.1 ± 7.4	0.3949
Number of cine runs until TAVR (min) ± SD	2.4 ± 1.4	1.6 ± 0.7	0.02
Post dilatation, n (%)	1 (1)	0 (0)	0.6643
Aortic regurgitation			
0, n (%)	78 (74)	14 (70)	0.9996
1, n (%)	25 (23)	6 (30)	
2, n (%)	2 (2)	0 (0)	
valve-in-valve (AI > 2), n (%)	2 (2)	0 (0)	0.1581
Device out of position	3 (3)	0 (0)	0.4483
**B Combined Outcome by VARC-2**			
**Outcome Data**	**Corhort A (n = 105)**	**Cohort B (n = 20)**	**p-value**
CPR, n (%)	2 (2)	0 (0)	0.5374
Vascular complications			
Minor vasc. complications, n (%)	26 (25)	2 (10)	0.1649
Major vasc. complications, n (%)	6 (7)	1 (5)	
Bleeding complications			
Life-threatening bleeding, n (%)	3 (3)	1 (5)	0.6593
Minor bleeding, n (%)	15 (14)	0 (0)	
Major bleeding, n (%)	2 (2)	0 (0)	
Acute kidney injury (Stage I-III) n (%)	12 (11)	6 (30)	0.2658
Need for dialysis, n (%)	4 (4)	1 (5)	0.8053
Myocardial infaction, n (%)	0 (0)	0 (0)	1.0000
Stroke, n (%)			
TIA, n (%)	0 (0)	0 (0)	0.5374
Severity grade 2 (ischemic")	2 (2)	0 (0)	
Need for pacemaker, n (%)	19 (18)	4 (20)	0.8423
Unplanned use of CABG, n (%)	0 (0)	0 (0)	1.0000
Other TAVR-related complications, n (%)	3 (3)	0 (0)	0.4483
30-day mortality, n (%)	3 (3)	0 (0)	0.4483

Values are mean ± SD or n (%); CPR = Cardiopulmonary resuscitation; CABG = cardiopulmonary

### Comparison of M-MSCT and A-MSCT for Prediction of Intraprocedural Annular Valve Angulation

#### Cohort A: Method-comparison

1. PPL-Analysis: The mean calculated PPL in LAO/RAO were +9.8±8.9 for M-MSCT, +12.2±7.8 for A-MSCT and +5.0±11.9 for CAA. The mean calculated PPL in CRAN/CAUD were -0.7±8.8 for M-MSCT, +1.8±8.2 for A-MSCT and -4.8±10.4 for CAA. There was a significant over-all difference related to independent chosen intraprocedural CAA (p<0.0001, please see Table 1).
2. Analysis of Automated vs. Manually Derived Parameters: The mean deviation of the PPL between M-MSCT and A-MSCT was similar in LAO/RAO (4.9±3.5) and CRAN/CAUD (5.1±3.6). Further analysis demonstrated that 100 cases (95%) of M-MSCT- and A-MSCT-predicted PPL in LAO/RAO and 99 cases (94%) in CRAN/CAUD were in accordance within 10° (Table 2).

The Bland–Altman analysis comparing M-MSCT and A-MSCT revealed a mean difference in LAO/RAO of 2.23° with limits of agreement between −9.42° and +13.87° and a significant strong correlation for predictions of the PPL in LAO/RAO (Fig 3, *r* = 0.76, p<0.0001). In CRAN/CAUD, M-MSCT and A-MSCT was also shown to have a strong correlation (*r* = 0.61, *p*<0.0001), with a mean difference of 2.43° and limits of agreement between −12.30° and +17.16°.

#### Cohort B: Clinical evaluation

1. PPL-Analysis: The mean calculated PPL in LAO/RAO were +13.0±9.6 for A-MSCT and +12.3±9.3 for CAA. The mean calculated PPL in CRAN/CAUD were +1.3±8.6 for A-MSCT and -2.8±6.7 for CAA. According to intraprocedural used preprocedural data of A-MSCT there was no significant difference in LAO/RAO (p = 0.8709) and CRAN/CAUD (p = 0.1474, please see Table 1) any more.
2. Accordance with Intraprocedural CAA: The mean deviation of the PPL was low comparing automated parameters (A-MSCT) to intraprocedural chosen CAA in LAO/RAO (2.8±2.0) and CRAN/CAUD (5.9±4.9), please see Table 2. The intraprocedural chosen CAA confirms the A-MSCT-predicted PPL with an accordance within 10° for 20 (100%) cases in LAO/RAO and in 17 (85%) cases in CRAN/CAUD (data not sown).

The Bland–Altman analysis comparing A-MSCT to CAA is depicted in the supplementary material (S2 Table, *r* = 0.94, p<0.0001). A significant, very strong, positive correlation was observed comparing A-MSCT to CAA for predictions of PPL in the LAO/RAO direction. A linear regression model revealed a medium but significant correlation in CRAN/CAUD when comparing A-MSCT to CAA.

#### Comparison between Cohort A and Cohort B

**Fig 4:**
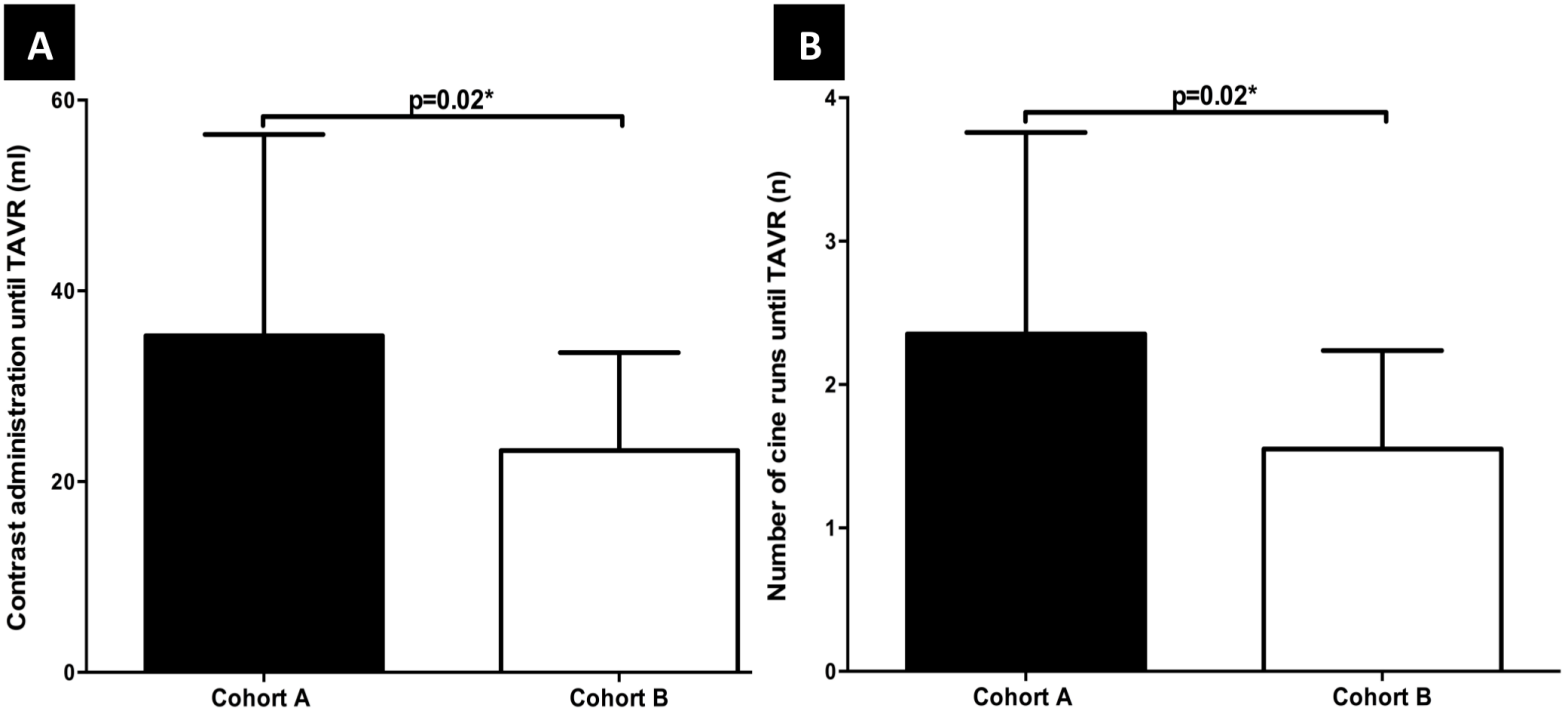
Clinical Outcome by Using the Preprocedural MSCT-datasets during Valve-Positioning. Preprocedural Use and Prediction of CAA is associated with a decrease in the amount of contrast agent use **(A)** and number of cine runs **(B)** until TAVR was reached.

Clinical evaluation: The amount of contrast (Cohort B: 23.3±10.3 ml, Cohort A: 35.3±21.1 ml, p = 0.02) and number of cine runs (Cohort B: 1.6±0.7 Loops; Cohort A: 2.4±1.4 Loops) to reach the preferred CAA were reduced by using the PPL information from preprocedural planning (Fig 4). Regarding further possible clinical benefits, there was no observation of moderate to severe aortic regurgitation, valve malposition or mortality after 30 days, using preprocedural calculated CAA for best deployment projection in Cohort B (Table 3).

## Discussion

The major findings of the present study are:

1. A fully automated segmentation tool reliably predicts the PPL and CAA for TAVR compared to parameters derived from an established manual segmentation tool.
2. The intraprocedural application of preprocedural fully automated MSCT-ST CAA-information reduces amount of contrast and number of cine runs during the TAVR procedure.
3. A software-guided CAA-approach is safe and efficient in self- and balloon-expandable prosthetic valves.

### The Role of MSCT in TAVR

Today, MSCT is the preferred modality for TAVR patient selection, planning and performance, providing information about anatomic conditions as well as the opportunity to reformat the reconstruction in any 3D-orientation [11–13]. Using CT-datasets manual segmentation tools have been developed to assess a perpendicular orthogonal view on the annulus valve plane [14–16]. To facilitate this process several automated and semiautomated, preprocedural MSCT-ST have been developed [14–19]. It could have been shown by interchangeability-analysis, that semiautomated aortic annulus evaluation is able to provide reliable results in the context of TAVR, with just minor correction by the user and allows more precise analysis in the hands of a less experienced reader [19]. But the safety and efficiacy of the regular application of an automated segmentation tool has not been analyzed in a “real world” clinical setting.

To investigate if there is suitable agreement between both methods, we used Bland-Altman analysis in which the results and differences were plotted against the average of the measurements obtained from the two methods. With the exception of a few outliers from the very poor MSCT quality, the Bland-Altman plot demonstrated that the limits of agreement are narrow, indicating that the methods are essentially equivalent. The most significant and strong correlation between M-MSCT and A-MSCT for predictions of the PPL was documented in the LAO/RAO direction.

### Role of the Aortic Annulus Plane in Fluoroscopy

For optimal catheter-based valve deployment, most physicians prefer a projection with all cusps in a planar line, wherein the central positioned RCC is symmetrically surrounded by the LCC and NCC. However, patient positioning during MSCT may not always agree with the positioning during TAVR, impeding the preprocedural prediction of the intraprocedural CAA. The angiographic assessment of the valvular plane, preventing overlap of the aortic root with the spine and descending aorta, presents another important limitation [20]. In our center, the majority of the TAVR procedures were performed with the self-expandable CoreValve system, requiring an angulation projection in which a distal radiopaque ring of the delivery catheter should appear closed in fluoroscopy. However, the distal radiopaque ring of the CoreValve device may not always appear well aligned even in the case of perfect aortic cusps alignment, particularly in horizontal and folded aortas. Taken together, several factors influence the final intraprocedural angulation. In Cohort B, adjustment of fluoroscopic angulation according to the preprocedural evaluation had an accordance of one hundred percent within 10 degrees in the LAO/RAO direction and 85% in the CRAN/CAUD direction. One study [16] demonstrated suitable angulations with accordance within four degrees in all spatial orientations in 84% of the patients. This study was restricted to the Edwards SAPIEN balloon-expandable prosthesis, obviating the need for further intraprocedural adjustment with respect to closure of the ring in the projection to the annular plane, as in the CoreValve system. Furthermore, all MSCTs were treated similarly according to “real world conditions” in a clinical set-up, and even under those conditions a remarkable accordance could have been demonstrated.

### Clinical Evaluation

We demonstrated that the intraprocedural use of CAA-informations gained by A-MSCT reduces amount of contrast during TAVR as well as the number of cine runs for establishing adequate PPL. Because the total intraprocedural amount of contrast medium and radiation time also depends on multiple angiographic presentations due to safety of vascular access, we additionally calculated the isolated amount of contrast medium and number of cine runs until preferred CAA was reached. It could have been repeatedly shown that the amount of contrast medium means an important risk factor for acute kidney injury after percutaneous coronary intervention and that minimizing the use of contrast volume remains one of the key challenges to improve periprocedural outcome in elderly and/or patients with preexisting CKD [21–22]. However, in this study the power to detect clinical relevant differences and to offer valide statements in respect of hard clinical endpoints are marginal due to the heterogenous sample-size.

### Limitations

We conducted an non-randomized trial with the risk of selection and ascertainment bias. Concerning the baseline assessment and the calculated p-values, it has to be considered that 5% of the tests will be subject to statistical type I error. However in our opinion, p-values of baseline assessments are still of some interest in estimation of potential differences between the groups which we could not namable detect.

The data in this study were based on a relatively small sample size in Cohort B to offer a valid statement on clinical outcome. The possible clinical benefits demonstrated in this study have to be investigated in prospective studies with more standardized proceedings and a more powered sample-size. It is known that the observed power is directly related to the p-value (the smaller the p-value the higher the power), so it is not categorial meaningful analysing the power to detect the difference actually observed. On the contrary, we are aware of the fact, that the two cohorts are not comparable with respect to clinical events.

Furthermore we offered no intra- and interobserver reproducibility because assessment of A-MSCT works fully automated and can’t be influenced by the operator himself. The analyses of M-MSCT and A-MSCT were performed independent from each other by 2 different and highly experienced operators, who did not were in consciousness of the counterpart and the related results in CAA-calculation.

## Conclusions

Our study showed that a fully automated segmentation tool is precisely able to predict corresponding perpendicular views and CAA as compared to parameters derived from a standard manual segmentation tool. We were able to demonstrate the safety and efficiacy of a software-guided CAA-approach in self- and balloon-expandable prosthetic valves for the first time. The automated system may help to perform valve implantation with reduced injection of contrast medium and radiation time and to improve short- and longterm outcome in patients with TAVR.

## Supporting Information

S1 File Protocol. (DOC)

S2 File Protocol (English). (DOCX)

S3 File Trend Checklist. (PDF)

S1 Table Patient Clinical and Functional Characteristics. (DOC)

S2 Table Correspondence in the MSCT-derived Prediction of the PPL and CAA. (DOC)

## Abbreviations

3D: 3-dimensional
CAA: C-Arm angulation
CRAN-CAUD: cranio-caudal
LAO: left anterior oblique
LCC: left coronary cusp
LVEF: left ventricular ejection fraction
MSCT: multislice computer tomography
MSCT-ST: MSCT-segmentation tool
A-MSCT: Automated derived CAA by MSCT
M-MSCT: Manual derived CAA by MSCT
NCC: noncoronary cusp
PPL: perpendicular line of the aortic annulus/perpendicular valve angulation(s)
TAVR: transcatheter aortic valve replacement
RAO: right anterior oblique
RCC: right coronary cusp
TA AVR: transapical aortic valve replacement
TF AVR: transfemoral aortic valve replacement
