# Supplementary material for: Comparison of Manual and Automated Preprocedural Segmentation Tools to Predict the Annulus Plane Angulation and C-Arm Positioning for Transcatheter Aortic Valve Replacement
Source: PLoS One. 2016 Apr 13;11(4):e0151918. doi: 10.1371/journal.pone.0151918 (PMC4830561; doi:10.1371/journal.pone.0151918)
Supplement: S1 File — (DOC) [file pone.0151918.s001.doc]

**Antrag zur Bewilligung eines Forschungsvorhabens durch die**

**Ethik-Kommission der Medizinischen Fakultät der**

**Heinrich Heine Universität Düsseldorf**

Register zur Multi-modalen Kardialen Bildgebung vor interventioneller Therapie von Klappenerkrankungen

**Institut:**

Klinik Kardiologie, Angiologie und Pneumologie

Direktor: Univ.-Prof. Dr. med. M. Kelm

Universitätsklinikum Düsseldorf

Moorenstraße 5

40225 Düsseldorf

**Hauptprüfer:**

Univ.-Prof. Dr. med. M. Kelm

**Koordination:** Rabea Wagstaff, B.Sc., M.A.

**Wissenschaftler:**

Dr. med. T. Zeus

Dr. med. R. Westenfeld

Dr. med. V. Schulze

Dr. med. J. Balzer

Dr. med. V. Veulmans

Dr. med. K. Hellhammer

Dr. med. F. Bönner

Dr. med. M. Neizel-Wittke

**Wissenschaftliche Kooperationspartner:**

Philips Healthcare, Philipsstraße 14, 20099 Hamburg

TomTec Imaging Systems, Edisonstraße 6, 85716 Unterschleißheim

Medtronic GmbH Earl-Bakken-Platz 1, 40670 Meerbusch

Edwards Lifescience Coorporation

**Inhaltsverzeichnis**

| 1) | Titel und wissenschaftliche Beschreibung | | 3 |
| --- | --- | --- | --- |
|  | Zusammenfassung | | 3 |
|  | Hintergrund | | 6 |
|  | Studienprotokoll | | 8 |
| 2) | Art der Studie | | 10 |
| 3) | Erklärung zu den Studiengrundsätzen | | 10 |
| 4) | Fallzahlabschätzung und vorgesehene Gesamtdauer | | 10 |
| 5) | Ein- und Ausschlusskriterien der Probanden | | 11 |
| 6) | Abbruchkriterien | | 11 |
| 7) | Mögliche unerwünschte Wirkungen von Medikamenten | | 11 |
| 8) | Komplikationen durch Untersuchungsmethoden | | 12 |
| 9) | Risiko-Nutzen-Abwägung | | 13 |
| 10) | Literaturverzeichnis | | 14 |
|  | |  | |
|  |  | |  |

**Abkürzungsverzeichnis**

AS Aortenstenose
CT Computertomographie
EF Ejektionsfraktion
HK Herzkatheter
KM Kontrastmittel
LV Linker Ventrikel
MACE Major Adverse Cardiac Event
MRT Magnetresonanztomographie
TAVI transkatheterielle Aorten-Klappen Implantation
TTE Transthorakale Echokardiographie
TEE Transösophgeale Echokardiographie
3D 3-dimensional

**1) Titel und wissenschaftliche Beschreibung**

**Multimodale Bildgebung vor interventioneller Klappentherapie**

**Zusammenfassung:**

Die Zahl degenerativer Klappenerkankungen des Herzens steigt in der alternden Bevölkerung der industrialisierten Länder dramatisch, wobei die Therapie der Aortenklappenstenose mit chirurgischen Klappenersatzverfahren von der Häufigkeit an erster Stelle steht. Viele dieser Patienten sind aufgrund ihrer Begleiterkrankungen jedoch mit den klassischen Verfahren nicht mehr operabel, so dass mit der Entwicklung einer perkutan kathetergesteuerten Implantation einer Bioklappe in dem letzten Jahrzehnt sich eine veritable Therapieoption eröffnet hat (1). Bei der transkatheter gesteuerten Aortenklappen Implantation (TAVI) wird über eine periphere arterielle Punktionsstelle (ca. 7 mm Durchmesser) der zusammengefaltete Klappenstent eingebracht und mittels Katheter-system in die Aortenklappenposition gebracht. Die erkrankte verengte Klappe wird mit einem Ballon an der Katheterspitze „aufgesprengt“ und die neue Bioprothese in der alten Klappenposition entfaltet und fest verankert. Diese neue Behandlungsmethode erscheint im Vergleich zur konventionellen Therapie mit Eröffnung des Brustkorps und operativem Ersatz der Herzklappe besonders schonend, da der Eingriff unter leichter Sedierung mit Erhalt der Spontanatmung durchgeführt werden kann und auf den Einsatz einer Herz-Lungenmaschine verzichtet wird. Die natürlichen Limitationen der perkutanen Klappenimplantation mit möglichen Komplikationsrisiken der Prothesendislokation und paravalvulären Leckage werden seit kurzem intensiv untersucht und zeigen bisher sehr positive Ergebnisse, welche die zukünftige Verbreitung des Therapieverfahrens unterstützen werden (2). Die prä-interventionelle Bildgebung ist von entscheidender Bedeutung für die Planung der Prozedur sowie Auswahl der korrekten Prothesengröße (3).


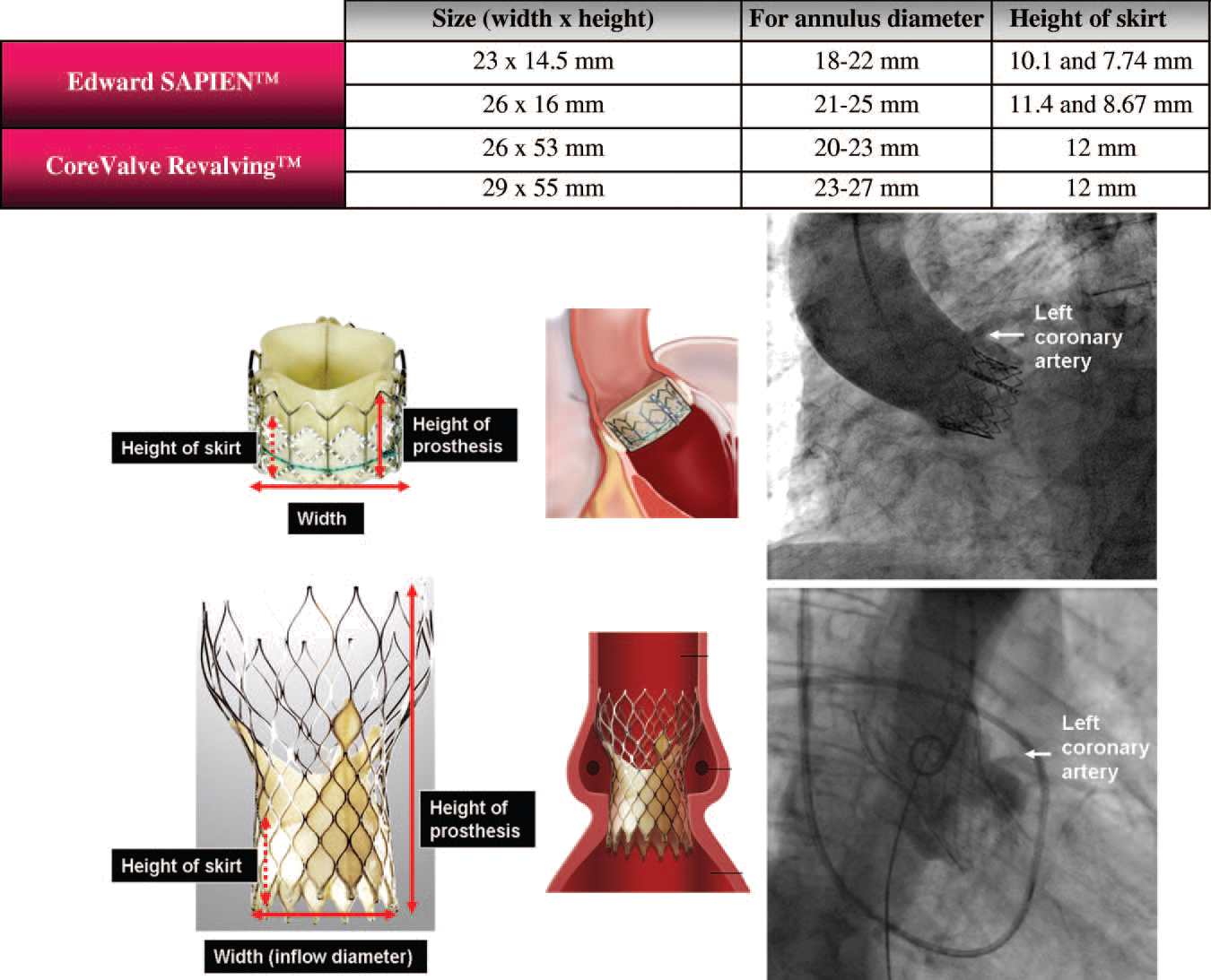


**Abbildung 1: Zugangsweg zur Klappenimplantation, Rechts: verschiedene Klappenprothesen und skizzierte Zielregion in der Aortenwurzel**

Ziel dieses Registers ist es, die Wertigkeit der verschiedenen Bildgebungsmodalitäten vor TAVI bezüglich der Patientenselektion, Planung und intraprozeduralen Steuerung der Klappenimplantation zu beurteilen. Die Daten sollen Informationen zu folgenden Fragestellungen liefern: Inwiefern sind die Messungen der verschiedenen Modalitäten miteinander vergleichbar? Inwiefern haben die Untersuchungsergebnisse der verschiedenen Bildgebungsmodalitäten Einfluß auf Patientenselektion, Wahl der Art der Prothese und Größe und Entscheidung des Zugangswegs? Läßt sich anhand der verschiedenen Bildgebungsmodalitäten Therapieerfolg und Hospitalisationsdauer nach Intervention ableiten?


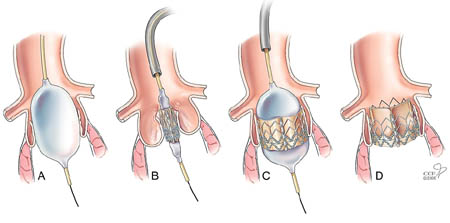


**Abbildung 2: Skizzierter Ablauf der Klappenimplantation mit vorhergehender Valvuloplastie**

Es werden in dieses Register Patienten aus den Jahren 2010 – 2014 mit symptomatischer Aortenklappenstenose, welche nach gemeinsamer Beurteilung von Kardiologen und Kardiochirurgen (Kardiochirurgisches Kolloquium) eine TAVI-Therapie erhalten sollen, aufgenommen.

Das peri-operative Risiko wird anhand verschiedener Scoring-Systeme gemeinsam mit der klinischen Evaluierung festgelegt. Alle Patienten erhalten folgende Voruntersuchungen unter Beachtung der jeweiligen Kontraindikationen wie im u.g. Schema (Abb.: 3) angezeigt. Patienten mit Therapieindikaton zur TAVI erhalten nach der Intervention primär echokardiographische Kontrolluntersuchungen zum Zeitpunkt der Entlassung, 1, 3 und 6 Monate nach der Intervention. Nach 6 Monaten werden nochmals ein 3D TEE und ein Kardio-MRT durchgeführt, um den Sitz der Klappenprothese sowie Ausmaß einer vorhandenen paravalvulären Regurgitation zu beurteilen. Ebenfalls können mit diesen Daten das myokardiale Remodeling nach Aortenklappenersatz beurteilt werden.

Primäres Ziel des Registers:

Primärer soll das Register Daten zum Prozedurerfolg gemessen an den Parametern: Interventionsdauer, korrekte Platzierung der Prothese, Ausmaß einer paravalvulären Regurgitation sowie Auftreten von intraprozedurale Komplikationen liefern.

Sekundäres Ziel des Registers:

Weiterhin soll das Register Informationen zur Mortalität und Hospitalisation aus kardialer/neurologischer Ursache im Beobachtungszeitraum geben. Hierbei werden als MACE jeder Myokardinfarkt, ein notfallmäßiger herzchirurgischer oder interventioneller Eingriff oder ein Schlaganfall gewertet.


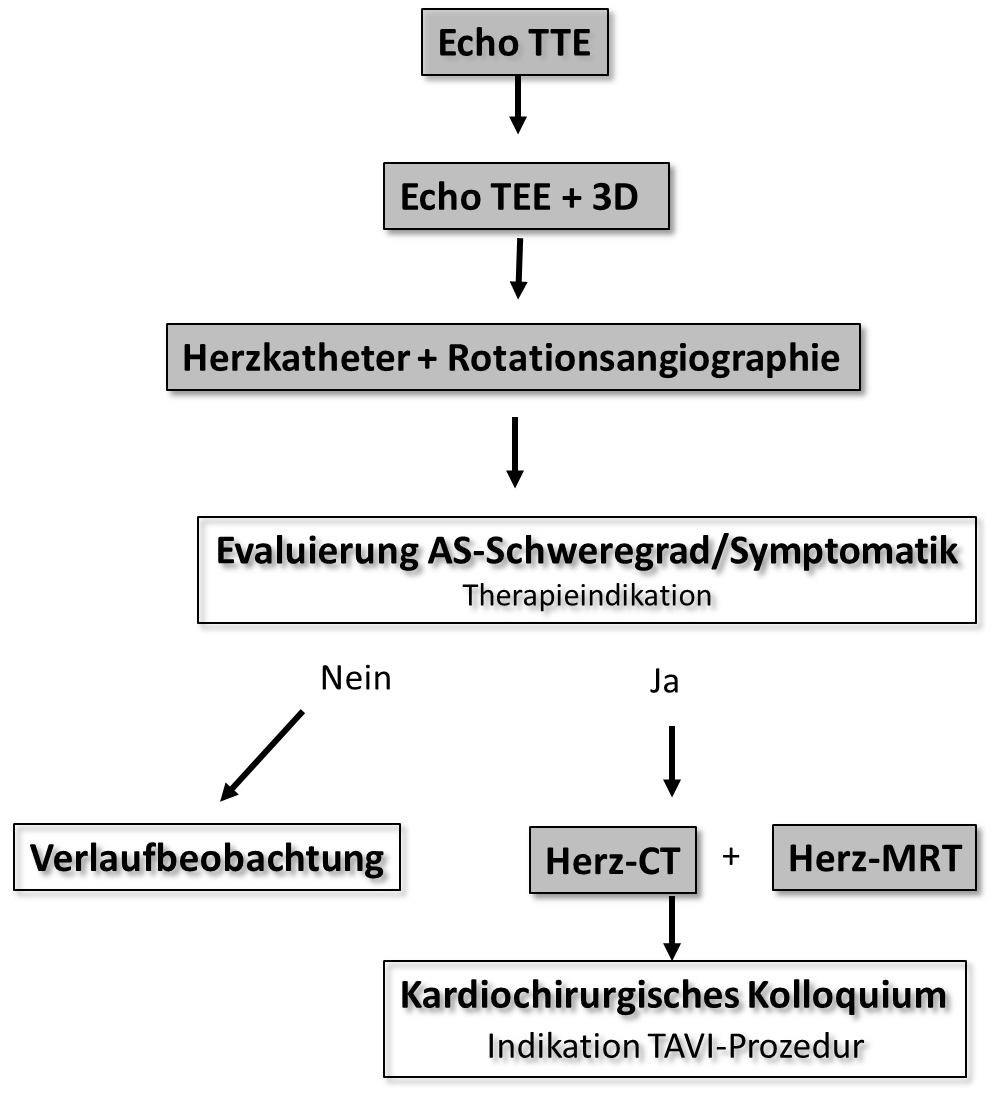


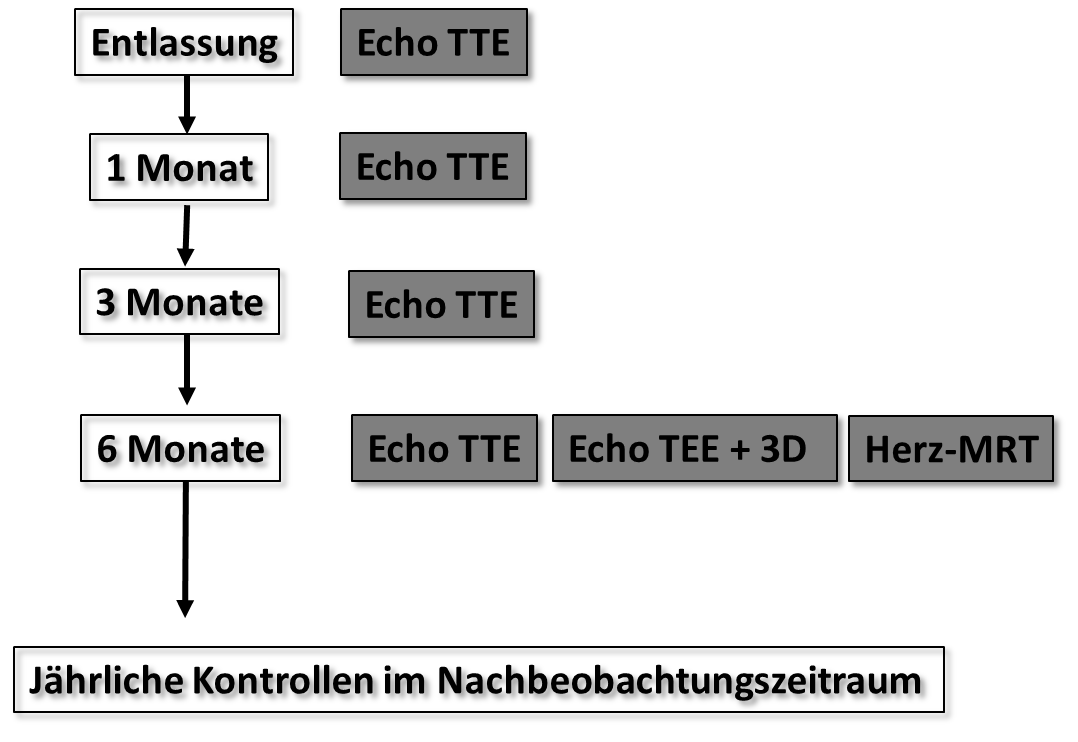


**Abbilung 3: Prozedurabfolge als Flußdiagramm**

**Hintergrund:**

Zur Risikostratifizierung sowie Planung der TAVI-Prozedur ist eine präinterventionelle Bildgebung zwingend erforderlich. Insbesondere die anatomische Abbildung der Aortenwurzel ist von essentieller Bedeutung, um die Diameter folgender Parametern zu bestimmen: Annulus, Bulbusweite, Sinutubulärer Übergang (STJ), Aorta ascendens (ao asc.), die Höhe der Ostienabgänge der Koronargefäße sowie die Länge der Klappentaschen. Letztere ermöglicht eine Risikoeinschätzung der Obstruktion der Koronarien während der Intervention. Hierneben werden als weniger standardisierte Parameter der Winkel zwischen linksventrikulärem Ausflußtrakt (LVOT) sowie der aufsteigenden Aorta gemessen. Zudem sind die Beurteilung der Aortenklappenverkalkung, der Gefäßverkalkung sowie das Ausmaß eines exzentrischen Gefäßverlaufs (Gefäßkinking) der thorakalen Aorta und der Beckenstrombahn von Bedeutung um den optimalen Zugangsweg und Art der Prothesenauswahl planen zu können. Gerade hinsichtlich der Auswahl der in verschiedenen Größen gefertigten Klappenprothese ist die Vermessung der Zielregion (insbesondere Annulus) der später implantierten Klappe von tragender Bedeutung. In verschiedenen Studien konnte schon gezeigt werden, dass erst dreidimensionale Schnittbildverfahren eine genaue Bestimmung des ellipsoiden Aortenannulus ermöglichen, wobei sich die Vermessung in der CT als "Goldstandard" etabliert hat (3-5). Im Gegensatz zu den modernen perkutanem Klappenersatzverfahren wird in der konventionellen Klappenchirurgie die Größenwahl der Klappenprothese am offenen Herzen mittels eines Messwerkzeugs ("Sizer") *in-situ* gemessen. Ein "Patienten-Prothesen Mismatch" kann neben erhöhten Druckgradienten vor allen Dingen in einer paravalvulären Leckage mit entsprechender Regurgitation resultieren, welche in neueren Studien als negative prognostische Prädiktoren identifiziert wurden. Zusätzlich zur Aortenwurzelanatomie ist die Beurteilung der globalen Herzgeometrie (enddiastolische/systolische Volumina und Diameter)mit den Funktionsparametern von Schlagvolumina und Strain-Analysen zur Myokarddeformation von Interesse, welche mittels Verfahren der Echokardiographie und kardialen MRT vor und nach Intervention erhoben werden.

Neben einer erfolgreichen Klappenimplantation ist gerade um die noch relativ neue Therapie kathetergestützter Klappenstents auch im Langzeitverlauf realistisch beurteilen zu können, die Erhebung dieser Basisparameter von Bedeutung (6).

In der Klinik für Kardiologie, Pneumologie und Angiologie werden im Jahr durchschnittlich ca. 300 Patienten für eine mögliche TAVI-Prozedur gesichtet und ca. 180 Klappenimplantationen jährlich durchgeführt. Der Screeningprozeß beinhaltet für eine optimale Prozedurplanung neben verschiedene Funktionsuntersuchungen und Labortests im Düsseldorfer Ansatz der multimodalen Bildgebung vor TAVI folgende Modalitäten: Echokardiographie mit 3D- Modalität, Herz-MRT, kardiale Computertomographie sowie Herzkatheteruntersuchung inklusive Rotationsangiographie der Aortenwurzel.

Die Echokardiographie ist eine kardiologische nichtinvasive Standarduntersuchung (Zeitdauer ca. 15 min). Mittels Ultraschall wird von den verschiedenen Herzachsen ein hochauflösendes Funktionsbild erstellt, um die muskuläre Pumpfunktion des Herzens sowie die Herzgeometrie zu beurteilen. Zudem kann eine erste anatomische Beschreibung der Klappenmorphologie erfolgen. Die Spektral-Doppleruntersuchung kann im Weiteren zur Quantifizierung der Klappenpathologie herangezogen werden. Zusätzlich werden drei-dimensionale Ultraschallverfahren eingesetzt, um eine möglichst exakte Visualisierung zur Vermessung o.g. Parameter zu ermöglichen. Mittels Strainanalysen werden im Weiteren quantitative Aussagen zur Myokarddeformation getroffen um mögliche Effekte der TAVI-Intervention zu untersuchen.

Das Herz-MRT ist eine relativ neue Bildgebungsmodalität in der Kardiologie, welche vergleichbar mit der Echokardiographie eine detaillierte Funktionsbeschreibung des Herzmuskels und Klappen ermöglicht. Es können zudem Fluss-Volumen quantifiziert werden und dreidimensionale Schnittbilder des Herzens erzeugt werden. Ein bedeutendes Alleinstellungsmerkmal hat die MRT bei der nichtinvasiven Darstellung von kardialem Narben/Fibrosegewebe mittels Spätkontrastaufnahmen (Late-Enhancement). Die Gesamtuntersuchungsdauer beträgt hierbei 60 min und jeweilige Kontraindikationen müssen hierbei beachtet werden, so scheiden sämtliche Träger eines ICD/SM-Aggregats im Patientenkollektiv von der Untersuchung aus. Anders als bei der Echokardiographie, bei der die Bildqualität sich interindividuell je nach Schallfenster und Ausmaß der Klappenverkalkung sehr unterscheidet, kann mit der MRT eine gleich hohe exzellente Bildqualität im Patientenkollektiv gewährleistet werden, weshalb diese Untersuchungsmethode gerade in der Verlaufsbeurteilung eine herausragende Bedeutung inne hat. Vergleichbar wie in der Echokardiographie können hier mittels Strainanalysen quantitative Aussagen zur Myokarddeformation erstellt werden.

Die kardiale Computertomographie ist ein etabliertes Verfahren, welches im Institut für diagnostische und interventionelle Radiologie an der MNR-Klinik durchgeführt wird (Untersuchungsdauer ca. 15 min). Hierbei werden nach Verabreichung eines 60-90 ml Kontrastmittelbolus (KM) unter einem Atemanhaltemanöver hochauflösende axiale Schnittbilder mit minimaler Schichtdicke in sehr kurzer Zeit akquiriert. Hierbei erfolgt eine EKG-Triggerung um die zyklische Herzbewegung zu kompensieren. Eine Funktionsanalyse des Herzens ist mit Hilfe dieser Aufnahmen nicht möglich und die Beurteilung der Anatomie auf eine gewählte Herzphase limitiert. Jedoch zeigen die Bilder eine exzellente räumliche Auflösung und dienen deshalb als "Goldstandard" bei der Vermessung eingangs genannter Parameter. Zusätzlich wird mit einer CT die Verkalkung der Klappen und großen Gefäße quantifiziert und die Morphologie der Klappenverkalkung qualitativ bewertet.

Die invasive Herzkatheteruntersuchung wird routinemäßig in der Klinik für Kardiologie vor anstehendem herzchirurgischen Eingriffen für die Beurteilung der nativen Gefäßversorgung sowie der hämodynamischen Parameter zum Schweregrad der Klappenerkrankung nach Einverständnis des Patienten durchgeführt. Vor geplantem Herzklappenersatz wird neben der arteriellen Links-Herzkatheteruntersuchung zudem eine venöse Rechtsherzuntersuchung durchgeführt, welche eine Beurteilung der Rechtsherzfunktion sowie weiteren hämodynamischen Parametern erlaubt. Im Weiteren wird mittels eines passageren Schrittmachers unter rapid ventricular pacing (RVP) die kontraktile Reserve des Herzens geprüft. Die Effektivität des RVP ist ein wesentlicher Bestandteil einer erfolgreichen Klappenimplantation, da hiermit der Auswurf aus dem Herzen minimiert wird und die Klappenprothese in korrekter Lage positioniert werden kann. Im Abschluss der Untersuchung wird eine Rotationsangiographie des Herzens unter Kontrastmittelgabe und RVP durchgeführt (Dauer ca. 5 sec). Hierbei vollführt der C-Bogen des Herzkatheters eine Propeller-Rotation um den Kopf des Patienten. Mit der Akquisition von verschiedenen Projektionen aus dem Rotationsverlauf wird mit dem großen Flat-Panel Detektor des C-Bogens eine Volumenaufnahme des Herzens generiert, welches im Nachgang zur Messung o.g. Parameter zur Verfügung steht.

Im Register „Düsseldorfer Modell der multimodeln Bildgebung vor TAVI“ sollen im ersten Schritt die Daten aus den verschiedenen, im Rahmen der TAVI-Vorbereitungsroutine angewandten Untersuchungen unter Berücksichtigung der entsprechenden Kontraindikationen, die o.g. Bildgebungstechniken zur exakten und vergleichenden Parametrierung der Aortenwurzel herangezogen werden. Jede der Bildgebungsmodalitäten zeigt spezifische Vor- und Nachteile auf, welche sich jedoch für eine optimale Prozedurplanung und Verlaufsbeurteilung nach Klappenimplantation additiv ergänzen.

Im Verlauf der Registerstudie sollen folgende Fragen beantwortet werden:

Haben 3D Echokardiographie, Herz-CT, Herz-MRT und Rotationsangiographie eine vergleichbare Sensitivität und Spezifität zur Beurteilung der Zielparameter in der Aortenwurzel zur Planung einer TAVI-Prozedur?

Welche Untersuchungsmodalitäten haben den höchsten prädiktiven Wert bezüglich der sekundären Endpunkte Hospitalisation und Mortalität nach TAVI-Prozedur im Patientenkollektiv?

**Studienprotokoll**

Das Register soll die diagnostische Wertigkeit der verschiedenen im Rahmen der TAVI-Vorbereitungsroutine angewandten Bildgebungsmodalitäten Echokardiographie, kardiale CT- und MRT-Untersuchung sowie Rotationsangiographie im Herzkatheter in Bezug auf die exakte Vermessung der Aortenwurzel miteinander vergleichen. Als weitere Untersuchungsparameter werden die geometrischen Herzvolumina sowie kardiale Funktionsparameter, Ejektionsfraktion in % und Strainwerte in der Echokardiographie und MRT erfasst.

Hierzu werden die Patienten mit hohem perioperativem Risiko (Euroscore >20%), welche nach gemeinsamer Besprechung im kardiochirurgischen Kolloquium zu einer TAVI-Prozedur vorgeschlagen wurden, nach Aufklärung und schriftlichem Einverständnis, in das Register aufgenommen. Die Untersuchungen TTE, 3D TEE, Herzkatheter mit Rotationsangiographie werden im ersten stationären Aufenthalt in der Klinik durchgeführt. Die Herz-CT bzw. Herz-MRT Untersuchungen werden poststationär ambulant durchgeführt, um die Untersuchungsbelastung für die Patienten zu vermindern und die Dauer des stationären Aufenthalts zu verkürzen. Die Durchführung der vorgenannten Bildgebungsmodalitäten ist bereits den diagnostischen Erfordernissen der TAVI-Prozedur folgend, fest in den klinischen Routineablauf implementiert.

Während der TAVI-Intervention wird die Prozedur mit Hilfe von Fuoroskopie (Röntgendurchleuchtung) auf dem Herzkathetertisch und ergänzend durch TTE/TEE gesteuert und das Prozedurergebnis überwacht. Vor Entlassung wird mit Echokardiographischen Verfahren das Ergebnis der Klappenimplantation sowie in den Nachkontrollterminen 1 und 3 Monate nach Intervention dokumentiert. Die Anzahl und Termine für die Ultraschalluntersuchungen entsprechen dem klinischen Standardprotokoll nach TAVI. Die 6 Monatskontrollen umfassen im Rahmen des Standardprotokolls neben der Echokardiographie, eine Herz-MRT Untersuchung, um die Anatomie der Aortenwurzel nach TAVI-Prozedur exakt zu evaluieren. Bei Vorliegen von Kontraindikationen (Schrittmacherimplantation) wird alternativ eine CT durchgeführt (siehe Abbildung 4 ).


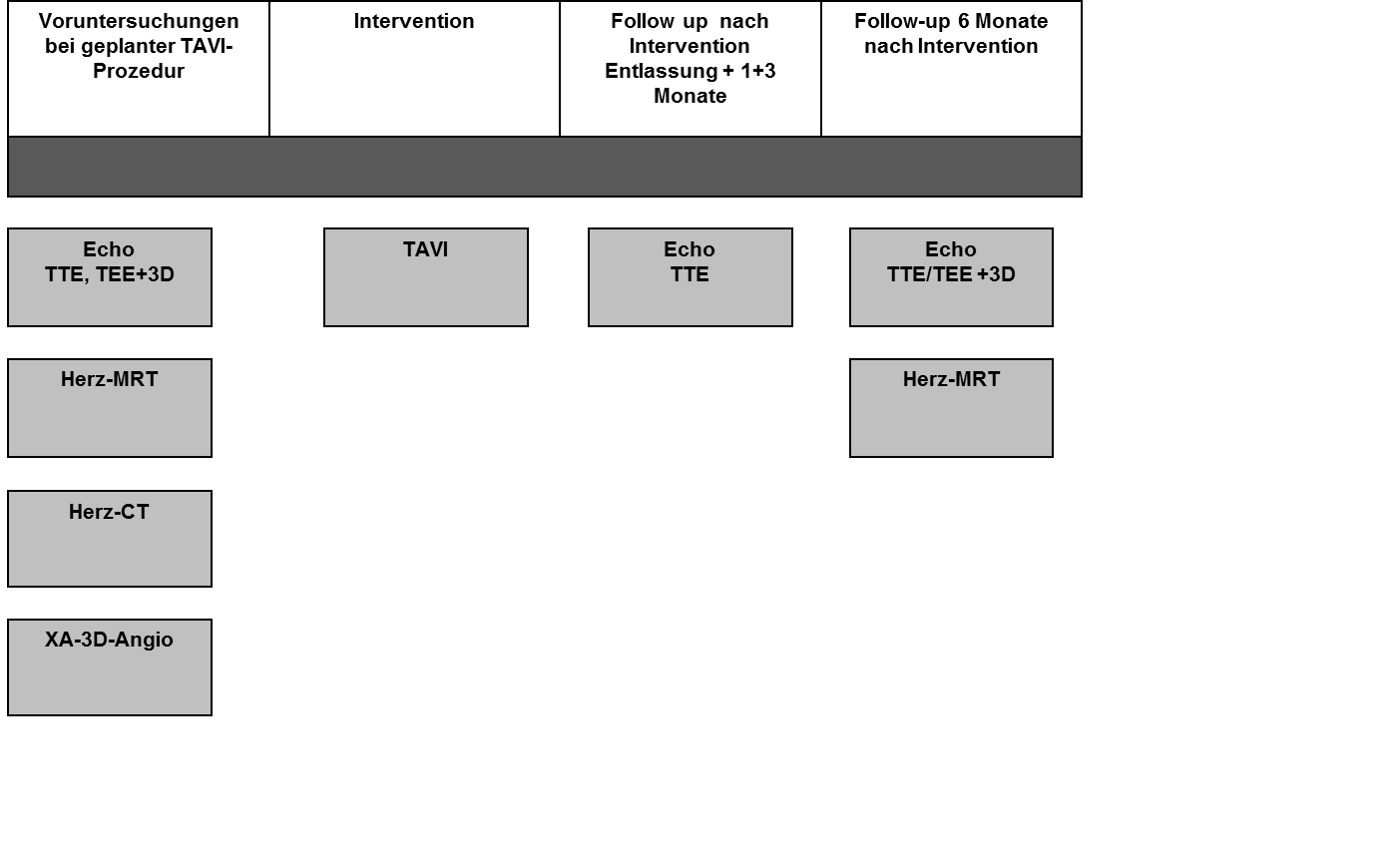


**Abbildung 4 Untersuchungsablauf**

Outcome: Zur Untersuchung des Outcomes werden die Datensätze von 2 unabhängigen, verblindeten in der klinischen Befundung erfahrenen Untersuchern ausgewertet. Im Weiteren erfolgt der Vergleich mit den intraprozeduralen Daten (Angulationswinkel des C-Arms zur Prothesenimplantation, Größe der Klappenprothese) mit den präprozeduralen Daten sowie die Daten aus dem Nachbeobachtungszeitraum.

Laborchemische Daten:

Neben den verschiedenen Bildgebungsmodalitäten möchten wir Routinelaborparameter vor und nach TAVI dokumentieren.

Routinelaborparameter: kleines Blutbild, Crea (ml/min), Harnstoff (mg/dl), GFR (ml/min), CRP (mg/dl)

Die routinemäßigen Blutabnahmen erfolgen im Rahmen der Voruntersuchung am Aufnahmetag, 1 Tag vor TAVI sowie an Tag 1 – 3 nach TAVI während sich der Patient in stationärer Behandlung befindet.

**2) Art der Studie**

Bei der Studie handelt es sich um eine retrospektive, monozentrische Registerstudie. Das Register soll Daten von Patienten aus den Jahren 2010 – 2020 erfassen, die das Merkmal einer therapiebedürftigen symptomatischen Aortenstenose haben und bei denen im kardiologisch-kardiochirurgischen TAVI-Kolloquium ein gemeinsamer Entschluß zum transkathetergesteuerten Aortenklappenersatz (TAVI) getroffen wurde.

Vor der geplanten Intervention werden nach o.g. Protokoll die verschiedenen Untersuchungsmodalitäten entsprechend des üblichen Standards durchgeführt und dokumentiert. Die in das Register aufgenommenen Daten werden gemäß ärztlicher Routine und unbeeinflusst in der Patientenauswahl, diagnostischer Maßnahmen oder therapeutischer Entscheidungen erhoben.

**3) Erklärung zu den Studiengrundsätzen**

Das Register soll die Daten von allen Patienten aus den Jahren 2010-2020 erfassen, für die aufgrund einer therapiebedürftigen Aortenstenose im kardiologisch-kardiochirurgischen TAVI-Kolloquium der Entschluss zum transkathetergesteuerten Aortenklappenersatz getroffen wurde und die nach erfolgter Aufklärung, ihr schriftliches Einverständnis zur Dokumentation und Auswertung ihrer Daten in dieses Register gegeben haben.

Eine Dokumentation und Weitergabe der Daten an wissenschaftliche Kooperationspartner erfolgt ausschließlich pseudonymisiert. Die Beachtung des Bundesdatenschutzgesetzes ist in vollem Umfang sichergestellt.

Da eine medikamentöse Behandlung kein Einschlusskriterium darstellt und keine medikationsspezifische Analyse geplant ist, unterliegt diese Registerstudie nicht den Regelungen des AMG. Alle verwendeten Geräte sind für die medizinische Anwendung genehmigt und unterliegen einer gesetzlich geregelten regelmäßigen Kontrolle. Die Registerstudie unterliegt somit nicht dem MPG, so dass weitere rechtliche Stellungnahmen entfallen.

**4) Fallzahlabschätzung und vorgesehene Gesamtdauer**

In diesem Register sollen von allen Patienten aus den Jahren 2010 - 2020 mit symptomatischer Aortenstenose mit der Indikation zum TAVI, die ihr schriftliches Einverständnis gegeben haben, Daten gesammelt werden. Alle Patienten die auf Grund der klinischen Symptomatik bzw. der Vorbefunde einer TAVI-Therapie zugeführt werden sollen, erhalten entsprechend der Routine eine 3D transösophageale Echokardiographie-, sowie nach Ausschluss von Kontraindikationen eine Herz-CT- und eine MRT-Untersuchung. Zudem wird bei allen Patienten eine Herzkatheteruntersuchung mit einer Rotationsangiographie der Aortenwurzel durchgeführt. Zur Zeit werden wöchentlich ca. 4-8 Patienten für eine mögliche TAVI-Prozedur in der Klinik für Kardiologie, Angiologie und Pneumologie am UKD gesichtet und gegebenenfalls einer entsprechend weiterführenden Diagnostik zugeführt. Im Jahr werden in Summe durchschnittlich 180-220 TAVI-Prozeduren durchgeführt. Der Nachbeobachtungszeitraum hinsichtlich Mortalität und Hospitalisation erstreckt sich über mindestens 36 Monate, erste Teilergebnisse jedoch nach 2 Jahren zu erwarten sind. Die Datenerfassung für das Register soll zunächst bis 2020 laufen.

**5) Ein- und Ausschlusskriterien**

*Einschlusskriterien für TAVI und Register:*

Patienten, die in das Register aufgenommen werden müssen die folgenden Kriterien erfüllen:

- Symptomatische, hochgradige Aortenklappenstenose
- Entscheidung des Herzteam für eine TAVI-Prozedur
- Bereitschaft und Fähigkeit am Register teilzunehmen
- Verständnis der Einverständniserklärung
- Schriftliche Einverständnisgabe zur Teilnahme am Register

*Ausschlusskriterien für das Register:*

- Fehlende Bereitschaft und Fähigkeit am Register teilzunehmen
- Fehlende Schriftliche Einverständnisgabe zur Teilnahme am Register

*Ausschlusskriterien für TAVI:*

- Schwerere Nierenfunktionsstörung (Glomeruläre Filtrationsrate < 30 ml/min/1,73m^2^)

*Ausschlußkriterien für die Herz-MRT:*

- Vorliegen von MR-Kontraindikationen (Standard MR-Aus­schluß­­kriterien):
- Elektrische Implantate wie Herzschrittmacher oder Perfusionspum­pen
- Ferromagnetische Implantate wie Aneurysmaclips, chirurgische Clips*,* Prothesen*,* künstliche Herzen, Klappen mit Metallteilen
- ausgeprägte Klaustrophobie

**6) Abbruchkriterien**

Abbruchkriterien für die beschriebene TAVI Prozedur sind ein Auftreten der unter Punkt 5 aufgeführten Ausschlusskriterien (Kontraindikationen fürs MRT, Klaustrophobie, Medikamentenunverträglichkeit, akute oder terminale Niereninsuffizienz).

Der Patient hat das Recht, zu jederzeit und ohne Angabe von Gründen, sein Einverständnis an dem Register zu wiederrufen, ohne dass ihm daraus Nachteile in seiner Behandlung entstehen.

**7) Mögliche unerwünschte Wirkungen durch Medikamente**

Es werden keine zusätzlichen Medikamente im Rahmen des Registers verabreicht.

Die im Rahmen der klinischen Routine verwendeten Kontrastmittel in der CT sowie Herzkatheteruntersuchung (Imeron -> CT; Accupaque-> HK; Bracco, Mailand, Italien) sind niederosmolare jodhaltige ionische Kontrastmittel (KM). Das Risiko einer kontrastmittelinduzierten Nephropathie wird in der Allgemeinbevölkerung mit 2% angegeben. Durch entsprechende Vorbereitung der Patienten und kontrastmittelsparenden Untersuchungsgang kann das Risiko verringert werden und durch einen verlängerten Nachbeobachtungszeitraum nach KM-Applikation das Auftreten von irreversiblen Nierenschädigungen minimiert werden. Weitere Nebenwirkungen sind vor allem Auftreten von Übelkeit und allergischen Reaktionen bis hin zur Anaphylaxie, welche jedoch sehr selten ist und bei frühzeitiger Therapie keine bleibenden Schäden hinterläßt. Potentiell besteht ebenfalls das Risiko einer KM-induzierten Schilddrüsenüberfunktion, so dass bei pathologischen Schilddrüsenfunktionsparametern vor Verwendung des Kontrastmittels eine entsprechende Vormedikation erfolgt.

Für die Herz-MRT Untersuchung ist die Gabe von MRT-Kontrastmittel nicht erforderlich. Prinzipiell wird für MRT-Untersuchungen gadoliniumhaltiges Kontrastmittel (Dotarem, Guerbet GmbH, Deutschland) verwendet. Bei schwer eingeschränkter Nierenfunktion (GFR<30 ml/min) kann die Verwendung von gadoliniumhaltigen Kontrastmittel zu der nephrogenen systemischen Fibrose führen, so dass eine Niereninsuffizienz diesen Ausmasses als Kontraindikation gilt. Bei nur leicht eingeschränkter Nierenfunktion sind keine anhaltenden Nebenwirkungen bekannt; Übelkeit oder allergische Reaktionen treten im Allgemeinen sehr selten bei MRT-Kontrastmitteln auf.

Das Sedativum Midazolam mit einer Halbwertzeit von 10-15 min für die TEE-Untersuchung ist sehr gut verträglich und ohne atemdepressive Wirkung. Vereinzelt zeigen sich paradoxe Medikamentenreaktionen jedoch nur von kurzer Dauer ohne medikamentösen Therapiebedarf und ohne anhaltende Langzeitfolgen.

**8) Komplikationen durch Untersuchungsmethoden**

Alle Untersuchungen, die in diesem Register dokumentiert werden, sind Standarduntersuchungsverfahren in der klinischen Routine und die Patienten werden über die Risiken und möglichen auftretenden Komplikationen einer jeden Untersuchung im einzelnen vorher schriftlich aufgeklärt.

Die kardiale CT ist eine risikoarme Untersuchungsmethode, welche durch prospektive EKG-Triggerung inzwischen mit geringer Strahlendosis hochauflösende Schnittbilder des Herzens anfertigt. Die obligate Gabe von ionischem Kontrastmittel kann hiermit assoziierte Nebenwirkungen hervorrufen (siehe Abschnitt 7, 9).

Bei der kardialen MRT handelt es sich um eine Routineuntersuchung. Bei den magnetresonanztomographischen Untersuchungen wird keine ionisierende Strahlung verwendet und die MRT-Untersuchung bringt nach heutigem Kenntnisstand keine bekannten oder vermuteten Nebenwirkungen mit sich.Die empfohlenen Richtwerte für die stationären und zeitlich veränderli­chen Magnetfelder, sowie für die Hochfrequenzfelder werden nicht überschrit­ten Patienten, die ein Kontraindikation für eine MRT-Untersuchung aufweisen (Defibrillator, etc.), werden nicht in das Register eingeschlossen.

Auch bei der Herzkatheteruntersuchung handelt es sich um eine Routineuntersuchung. Allgemein kommen in weniger als einem Prozent aller Herzkatheter-Untersuchungen Komplikationen vor. Dazu gehören Herzrhythmusstörungen, Herzinfarkt, Embolien, Thrombose, Überempfindlichkeits-Reaktionen, Infektionen, Gefäßverletzungen und Blutungen bei der Punktion bzw. Bluterguss nach der Punktion, Haut-, Weichteil- und Nervenverletzungen.

Für die Erfassung der Routinelaborparameter erfolgt keine zusätzliche Blutabnahme. Zu den üblichen Risiken der Blutabnahme gehört das Entstehen von Hämatomen im Bereich der Einstichstelle. Es besteht das sehr geringe Risiko einer lokalen oder allgemeinen Infektion. In extrem seltenen Fällen kann es zu einer Verletzung eines Hautnervs, evtl. sogar mit chronischem Verlauf, kommen.

**9) Risiko-Nutzen-Abwägung**

Da alle Untersuchungen zum routinemäßig eingesetzten Armentarium für die Diagnose der Aortenklappenstenose und weiteren Entscheidung des Therapiewegs gehören, entstehen für die in das Register aufgenommenen Patienten keine zusätzlichen Risiken.

**Risiko-Nutzen-Abwägung des Standarduntersuchungsverfahrens:**

Die Echountersuchungen sind mit keinen Nebenwirkungen verbunden. Die Sedierung für die TEE-Untersuchung (Schluckecho) ist gut verträglich und Nebenwirkungen bei Medikamentenunverträglichkeit sehr selten und nur von vorrübergehender Dauer (paradoxe Medikamentenreaktion mit psychischem Durchgangssyndrom). Die Herz-MRT ist ein bildgebendes Verfahren ohne ionisierende Strahlung. Bei Einhaltung der Kontraindikationen (ferromagnetische Implantate) besteht kein relevantes Untersuchungsrisiko. Die Herz-CT-Untersuchung ist mit modernen prospektiv EKG-getriggerten Verfahren gering in der Strahlendosis (8-12 mSv), jedoch ist die Verwendung von Kontrastmittel obligat mit den verbundenen potentiellen Nebenwirkungen. Die Herzkatheteruntersuchung mit Darstellung der Koronargefäße und Rotationsangiographie sowie invasiven intrakardialen Druckmessungen hat ebenfalls eine vergleichbar geringe Strahlendosis von 3-4 mSv. Das Risiko eines akuten Nierenversagens kann bei Verwendung von Kontrastmittel durch entsprechende Vorbereitung und Nachbeobachtung minimiert werden, ist jedoch aber grundsätzlich bei Verwendung von ionischem Kontrastmittel nicht gänzlich auszuschließen. Das Risiko wird in der Allgemeinbevölkerung mit ca. 2% angegeben.

Ein routinemäßiger Nachbeobachtungszeitraum von vier Jahren ist festgelegt.

**10) Literaturverzeichnis:**

1. Makkar RR, Fontana GP, Jilaihawi H, Kapadia S, Pichard AD, Douglas PS, et al. Transcatheter aortic-valve replacement for inoperable severe aortic stenosis. The New England journal of medicine 2012;366:1696-1704.
2. Kodali SK, Williams MR, Smith CR, Svensson LG, Webb JG, Makkar RR, et al. Two-year outcomes after transcatheter or surgical aortic-valve replacement. The New England journal of medicine 2012;366:1686-1695.
3. Messika-Zeitoun D, Serfaty JM, Brochet E, Ducrocq G, Lepage L, Detaint D, et al. Multimodal assessment of the aortic annulus diameter: implications for transcatheter aortic valve implantation. Journal of the American College of Cardiology 2010;55:186-194.
4. Ng AC, Delgado V, van der Kley F, Shanks M, van de Veire NR, Bertini M, et al. Comparison of aortic root dimensions and geometries before and after transcatheter aortic valve implantation by 2- and 3-dimensional transesophageal echocardiography and multislice computed tomography. Circulation Cardiovascular imaging 2010;3:94-102.
5. Jilaihawi H, Kashif M, Fontana G, Furugen A, Shiota T, Friede G, et al. Cross-sectional computed tomographic assessment improves accuracy of aortic annular sizing for transcatheter aortic valve replacement and reduces the incidence of paravalvular aortic regurgitation. Journal of the American College of Cardiology 2012;59:1275-1286.
6. Tamburino C, Capodanno D, Ramondo A, Petronio AS, Ettori F, Santoro G, et al. Incidence and predictors of early and late mortality after transcatheter aortic valve implantation in 663 patients with severe aortic stenosis. Circulation 2011;123:299-308
