# Supplementary material for: Comparison of Manual and Automated Preprocedural Segmentation Tools to Predict the Annulus Plane Angulation and C-Arm Positioning for Transcatheter Aortic Valve Replacement
Source: PLoS One. 2016 Apr 13;11(4):e0151918. doi: 10.1371/journal.pone.0151918 (PMC4830561; doi:10.1371/journal.pone.0151918)
Supplement: S2 File — (DOCX) [file pone.0151918.s002.docx]

Ethics application to institutional Ethics Committee of the Heinrich-Heine University

Multi Modal Cardiac Imaging Prior Transcatheter Aortic Valve Implantation

**Institute:**

Division of Cardiology, Pulmonary Diseases, Vascular Medicine University Hospital Düsseldorf

Professor and Chair: Univ.-Prof. Dr. med. M. Kelm

Moorenstr. 5

40225 Düsseldorf, Germany

**Principal investigator:**

Univ.-Prof. Dr. med. M. Kelm

**Coordination:** Rabea Wagstaff, B.Sc., M.A.

**Investigators:**

Dr. med. T. Zeus

Dr. med. R. Westenfeld

Dr. med. V. Schulze

Dr. med. J. Balzer

Dr. med. V. Veulemans

Dr. med. K. Hellhammer

Dr. med. F. Bönner

Dr. med. M. Neizel-Wittke

**Investigative Cooperations:**

Philips Healthcare, Philipsstraße 14, 20099 Hamburg

TomTec Imaging Systems, Edisonstraße 6, 85716 Unterschleißheim

Medtronic GmbH Earl-Bakken-Platz 1, 40670 Meerbusch

Edwards Lifescience Coorporation

**Table of contents**

| 1) | Title and investigative work | 3 |
| --- | --- | --- |
|  | Summary | 3 |
|  | Background | 7 |
|  | Study protocol | 9 |
| 2) | Type of study | 11 |
| 3) | Statement on the principles of the study | 11 |
| 4) | Sample size estimation and intended duration | 11 |
| 5) | Inclusion and exclusion criteria | 12 |
| 6) | Termination criteria | 12 |
| 7) | Possible side effects of medication | 12 |
| 8) | Procedure-related complications | 12 |
| 9) | Risk-benefit analysis | 13 |
| 10) | Bibliography | 14 |
|  |  |  |

**Abbrevation list**

AS Aortic Stenosis
CT Computer Tomography
EF Ejektion Fraction
CM Contrast Media
LV Left Ventricle
MACE Major Adverse Cardiac Event
MRI Magnetic Resonance Imaging
TAVI Transcatheter Aortic Valve Implantation
TTE Transthoracic Echokardiography
TEE Transoesophgeal Echokardiography
3D 3-dimensional

**1) Title and investigative work**

**Multimodale imaging prior Transcatheter Aortic Valve Implantation**

**Summary:**

The incidence of degenerative valve diseases dramatically increases in the aging population of the industrialized countries. Among the valve diseases aortic stenosis is the one most frequently treated. Many of the patients, though, are not operable because of various [comorbidities](http://www.linguee.de/englisch-deutsch/uebersetzung/comorbidities.html). Thus, the percutaneous chatheter-derived implantation of a biological valve has become a veritable therapeutic option during the last decade(1).

During Transcatheter Aortic Valve Implantation (TAVI) a catheter holding the compacted replacement valve is inserted into a peripheral artery and positioned within the aortic valve level. The catheter-system also contains a balloon that pushes the leaflets of the native valve aside and then anchores the new valve within the old one. This new therapy is quite gentle compared to the conventional open-chest surgery, as the implantation can be performed without general anaesthesia or a life support machine. The natural limitations of the percutaneous valve replacement (i.e. complications like device dislocation or paravalvular leakage) have lately been objects of thorough investigation. Until now the results seem rather promising, which will support the further expansion of the technique (2). The pre-interventional imaging is crucial for the planning of the procedure and the correct choice of the device (3).


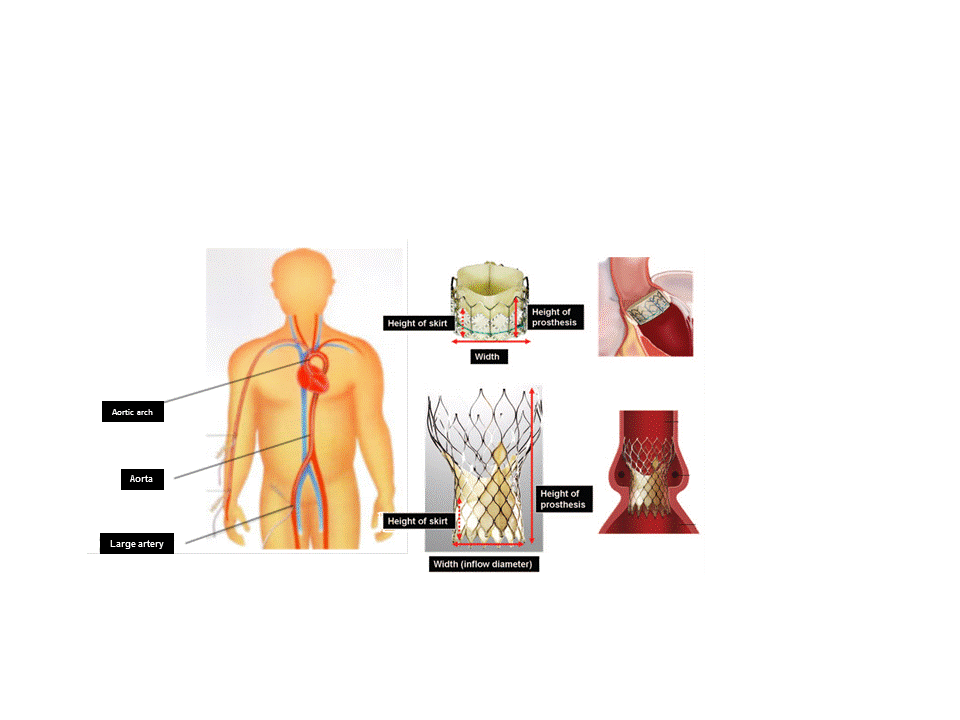
**Figure 1: Access path to valve implantation (left); different valve prostheses and sketched target region in the aortic root (right)**

The objective of the study is the evaluation of the different imaging modalities concerning patient selection, preprocedural planning and intraprocedural monitoring.

The data should answer the following questions: In what way can the different imaging techniques and the particular measurements be compared to one another? Do the findings influence the patient selection, the choice of device and of access? Does the use of different imaging modalities affect the [therapeutic success](http://www.linguee.de/englisch-deutsch/uebersetzung/therapeutic+success.html) and the average number of hospital days after intervention?


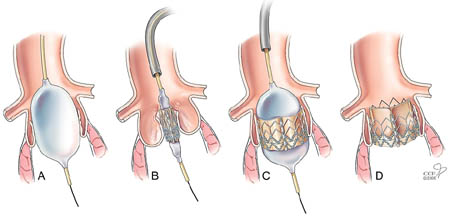


**Figure 2: Sketch of a valve implantation with previous balloon valvuloplasty**

The registry includes all patients, who presented with a severe and symtomatic aortic stenosis in 2010-2014 and who qualified für TAVI procedure in accordance with a joint decision of both cardiologists and cardiac surgeons (heart team).

The peri-operative risk is evaluated using varius scoring systems in addition to the clinical assessment. All patiens undergo the examinations (as far as not contraindicated) presented in Figure 3. After TAVI procedure the patients receive echokardiographic controls at the time ofdischarge, and one, three and six months post procedure. Six months after intervention a 3D TEE and cardiac MRI is peformed in order to evaluate the fit of the prothesis and the extent of the paravalvular leakage. In addition, the myocardial remodeling can be examined.

Primary objective of the registry:

The study should supply data concerning the success of the procedure. The parameters are: duration of the procedure, correct placement of the prothesis, extent of paravalvular leakage and intraprocedural complications.

Secondary objective of the registry:

Moreover information on mortality and hospitalization due to cardiac/neurological cause during the observation period should be acquired. MACE is defined as any myocardial infarction, emergency heart surgery/intervention or stroke.


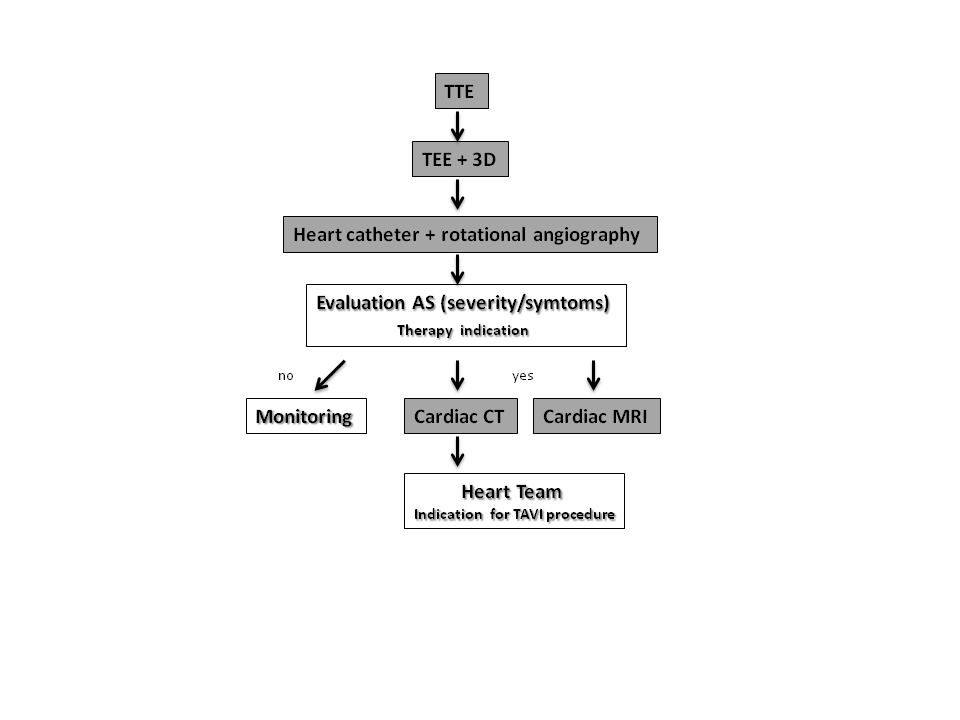


**
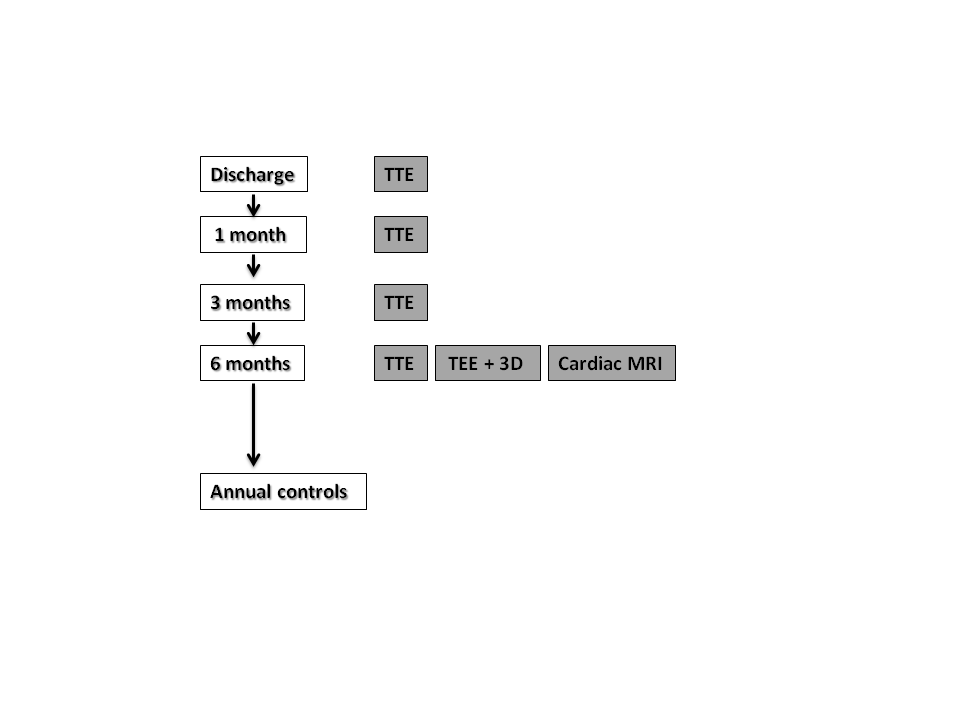
**

**Figure 3: Order of examinations as a flow chart**

**Background:**

Preinterventional imaging is necessarily required for risk stratifikation and planning of the TAVI procedure. Especially the mapping of the aortic root is essential to determine the diameters of the following structures: annulus, sinus of valsalva, sinutubular junction (STJ), aorta ascendens (ao. asc.), coronary height and length of the cusps. The latter allows a risk assessment of coronary obstruction during the procedure. In addition to this, the angle between the left ventricular outflow tract (LOVT) and the aorta serves as a predictor for coronary obstruction, although it is a less standardized parameter.

Moreover, [it](https://dict.leo.org/ende/index_de.html#/search=evaluation&searchLoc=0&resultOrder=basic&multiwordShowSingle=on) is crucial to evaluate the calcification of the aortic valve and the vessels as well as the extent of the kinking of the vessels in order to choose the correct interventional access and prothesis. The sizing of the target region (especially the annulus) is very important regarding the different dimensions of the valve protheses. Various studies revealed that only three dimensional tomographic imaging techniques allow an exact sizing of the ellipsoid aortic annulus. The CT has been established as the gold standard (3-5). In contrast to transcatheter valve implantations cardiac surgeons determine the size of the valve graft *in situ* using a [measuring tool](http://www.linguee.com/english-german/translation/measuring+tool.html) („sizer“) on the open heart. A “patient-prothesis-mismatch“ can result in elevated pressure gradients and in paravalvular leakage with suitable regurgitation, which new studies identified as negative prognostic factors.

In addition to the anatomy of the aortic root it is interesting to look at the global cardiac geometry (enddiastolic/sytolic volumina and diameters) and the functional parameters (stroke volumina and strain analyses). These information can be obtained using echocardiography and MRI before and after the intervention.

The assessment of these baseline parameters is not only crucial to successfully perfome a TAVI procedure, but to evaluate the long-time development of this relatively new therapy(6).

In the Division of Cardiology, Pulmonary Diseases and Vascular Medicine every year 300 patients are screened for potential TAVI and 180 procedures are performed. The screening includes various functional and laboratory tests, as well as multimodal imaging techniques in order to optimize the TAVI procedure: 3D echocardiography, cardiac MRI, cardiac CT and heart catheterization including rotatational angiography of the aortic root.

Echokardiography is a cardiological, non-invasive standard test (expenditure of time: 15 minutes). Ultrasound is used to create a high-definition functional image of several cardiac axes. Thus, the pump function and the geometry of the heart are evaluated. Moreover, one can gain a first impression of the valve morphology. For further quantification of the valve pathology spectral doppler imaging is used. In addition, 3D echokardiography allows an almost exact visualization of the valve. One can get information of the parameters namend-above from these 3D pictures. Strain analyses enable quantification of myocardial deformation in order to evaluate possible effects of the TAVI procedure.

Cardic MRI is a relatively new technique in cardiology and is comparable to echocardiography as it provides detailed functional informations of the heart muscel and the valves. Additionally, it generates three dimensional tomographic images and allows flow-volume quantification.

The unique feature of the MRI is the non invasive visualization of cardiac scars/fibrotic tissue using late enhancement imaging. The expenditure of time is about 60 minutes and there are some contraidications: patients with [implantable cardioverter-defibrillators](https://en.wikipedia.org/wiki/Implantable_cardioverter-defibrillator) /pacemaker are excluded. In contrast to echocardiography, where the quality can vary interindividually due to acoustic windows or severe calcification of the valve, MRI imaging supplies a solid quality. This is why MRI imaging is so important to observe the development after the implantation. Like strain analyses in echocardiography MRI can provide quantative information on myocardial deformation.

Multi-slice computer tomography (MS-CT) is a well-established method, which is executed by the Institute for diagnostic and interventional Radiology of the University Hospital Düsseldorf (expenditure of time: 15 minutes). With this technique, axial high-definition images can be acquired in a short period of time (slice thickness < 1mm). There is need of 60-90 ml of contrast media and the patient carrying out breathing maneuvers. In order to compensate the cyclic heart movement the examination is ECG-triggered. It is not possible, though, to analyze functional parameters and the evaluation of the anatomy is limited to the reconstruction of a selected phase. Nonetheless, MS-CT provides images with high spatial resolution and has, thus, become the „gold standard“. In addition, CT images supply information on the calcification of the aortic valve and the vessels.

Fluoroscopy is routinely performed in the Divison of Cardiology ahead of cardiosurgical procedures in order to explore the coronary arteries and the valves. Ahead of a scheduled valve replacement a venous right heart catheterization is performed as well, as it provides information on the right heart function and hemodynamic parameters. Moreover, the myocardial contractile is tested with rapid ventricular pacing (RVP) via a temporary pacemaker. Effective RVP is crucial for a successful TAVI, as it minimizes the cardiac output while the prothesis is correctly positoned. Finally, a rotational angiography is performed under administratioin of contrast agent and RVP (5 sec). The c-arm carries out a propelling rotation around the patient’s head. Thus, the flat-panel detector of the c-arm constructs volume images of the heart, which can be used for measuring hereafter.

The registry „Multi Modal Cardiac Imaging Prior Transcatheter Aortic Valve Implantation“ will contain data derived from the various imaging tests routinely performed as part of the TAVI screening.

The first step is to use these information to measure the aortic root and to compare the diferent imaging techniques in respect to these measurements. Every imaging technique has its specific power, that contributes to an optimal planning of the procedure and follow-up.

Furthermore, there are the following questions to be answered:

1. Do 3D echocardiograpy, cardiac CT, cardiac MRI and rotational angiography have comparable sensitivity and specificity regarding correct measurement of the aortic root?
2. Which imaging technique has the highest predictive value in respect to the secondary endpoints (hospitalization and mortality after TAVI procedure)?

**Study protocol**

The registry is supposed to compare the diagnostic values of different imaging techniques (echocardiograpy, cardiac CT, cardiac MRI and rotational angiography) with regard to correct sizing of the aortic root. In addition, parameters like geometric heart volumina, ejection fraction (%) and strain analyses (MRI/echocardiography) are acquired.

Patients, who are mutually accepted for TAVI by cardiologists and cardiac surgeons (heart team) because of their high perioperative risk (log euroscore > 20%), are included in the study after obtaining informed consent.

TTE, 3D TEE and fluroscopy including rotational angiography are scheduled during the first stay in hospital. The tests are already implemented in daily routine. MS-CT and cardiac MRI are performed as outpatient procedures to reduce time of hospitalization and stress for the patient.

The TAVI procedure is monitored by fluoroscopy and TTE/TEE in addition. Prior discharge as well as 1 and 3 months after the intervention TTE is performed as follow-up. The number and scheduling of follow-ups accord to the standard TAVI protocol. 6 months after TAVI procedure a cardiac MRI is performed as well to evaluate the anatomy of the aortic root. If contraindicated (pacemaker) a CT is performed instead (Figure 4 ).

Outcome:

The datasets were analyzed by two independent, blinded and trained investigators. Furthermore the intraprocedural data (angulation of the c-arm during implantation, size of the valve prothesis) were compared with pre- and postprocedural date.


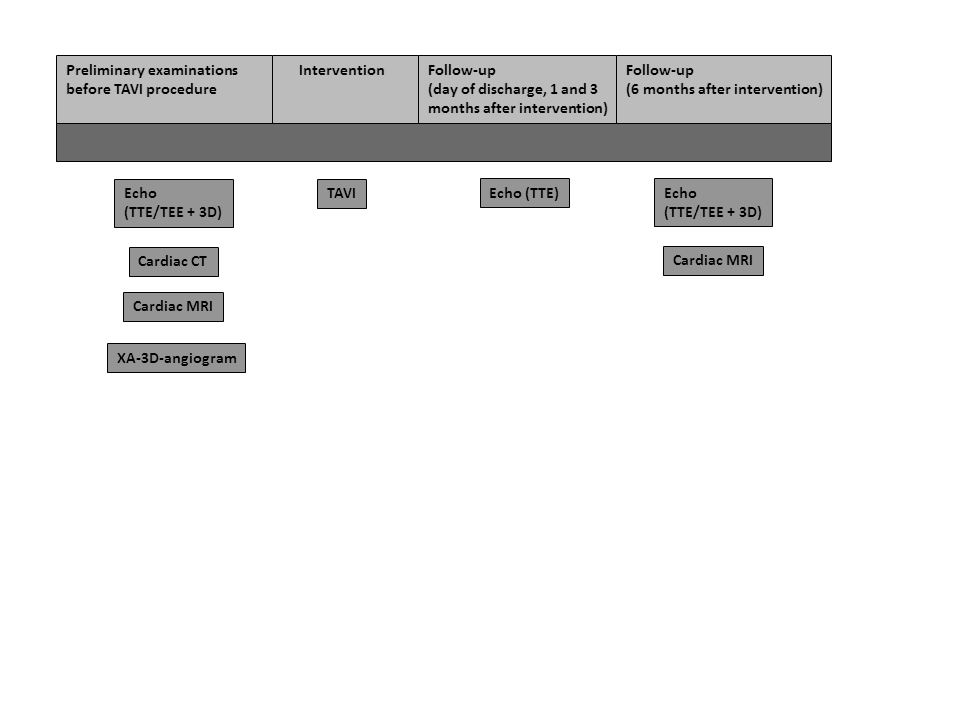


**Figure 4 Exmination workflow**

Laboratory results:

Next to imaging and functional tests the following laboratory tests were accomplished.

Routine: blood cell count, creatinine (mg/dl), urea (mg/dl), GFR (ml/min), CRP (mg/dl)

The blood was taken on the day before TAVI and on day 1-3 post TAVI.

**2) Type of study**

The study is a retropective, monocentric registry study. It is supposed to collect data from 2010-2020. Patients have to meet the criterion of a symptomatic severe aortic stenosis with need for treatment. Moreover, the patient has to be accepted for TAVI by cardiologists and cardiac surgeons (Heart Team). Prior to intervention the examinations named-above are carried out and documented in a standardized manner.

**3) Statement on the principles of the study**

The study includes data of patients with a severe aortic stenosis (all-comers 2010-2020), who are accepted for TAVI procedure by the Heart Team and who give informed consent on documentation and analysis of their data.

Documentation and passing-on to fellow cooperation partners is only permitted in an pseudominized way. The study is carried out in strict compliance with the Bundesdatenschutzgesetzes (Federal Data Protection Act). As drug therapy is no inclusion criterion and as no drug specific analysis is planned, this study is not subject to the Arzneimittelgesetz (Medicines Law). All devices used are approved for medical use and are subject to a periodic inspection. The registry study is therefore not subject to the Medizinproduktegesetz (medical products law), so that there is no need for further legal opinions.

**4) Sample size estimation and intended duration**

This study will include data from all patients from the years 2010 - 2020 with symptomatic aortic stenosis with the indication for TAVI, who have given their written consent. Patients, who are likely to be accepted for TAVI because of clinical assessment and preliminary findings, receive a routine transesophageal echocardiography 3D and (after exclusion of contraindications) a cardiac CT and an MRI scan. In addition, in all patients a cardiac catheterization is performed with a rotational angiography of the aortic root. Currently about 4-8 patients a week are screened for a possible TAVI procedure in the Division of Cardiology, Angiology and Pneumology. An average of 180-220 TAVI procedures are performed in total every year. The follow-up with regard to mortality and hospitalization extends over at least 36 months, the first partial results, however, are to be expected after 2 years. The data collection for the registry is intended to run until 2020.

Synonyme für In diesem Register sollen von allen Patienten aus den Jahren 2010 - 2020 mit symptomatischer Aortenstenose mit der Indikation zum TAVI, die ihr schriftliches Einverständnis gegeben haben, Daten gesammelt werden. Alle Patienten die auf Grund der klinischen Symptomatik bzw. der Vorbefunde einer TAVI-Therapie zugeführt werden sollen, erhalten entsprechend der Routine eine 3D transösophageale Echokardiographie-, sowie nach Ausschluss von Kontraindikationen eine Herz-CT- und eine MRT-Untersuchung. Zudem wird bei allen Patienten eine Herzkatheteruntersuchung mit einer Rotationsangiographie der Aortenwurzel durchgeführt. Zur Zeit werden wöchentlich ca. 4-8 Patienten für eine mögliche TAVI-Prozedur in der Klinik für Kardiologie, Angiologie und Pneumologie am UKD gesichtet und gegebenenfalls einer entsprechend weiterführenden Diagnostik zugeführt. Im Jahr werden in Summe durchschnittlich 180-220 TAVI-Prozeduren durchgeführt. Der Nachbeobachtungszeitraum hinsichtlich Mortalität und Hospitalisation erstreckt sich über mindestens 36 Monate, erste Teilergebnisse jedoch nach 2 Jahren zu erwarten sind. Die Datenerfassung für das Register soll zunächst bis 2020 laufen.

Beispiele für In diesem Register sollen von allen Patienten aus den Jahren 2010 - 2020 mit symptomatischer Aortenstenose mit der Indikation zum TAVI, die ihr schriftliches Einverständnis gegeben haben, Daten gesammelt werden. Alle Patienten die auf Grund der klinischen Symptomatik bzw. der Vorbefunde einer TAVI-Therapie zugeführt werden sollen, erhalten entsprechend der Routine eine 3D transösophageale Echokardiographie-, sowie nach Ausschluss von Kontraindikationen eine Herz-CT- und eine MRT-Untersuchung. Zudem wird bei allen Patienten eine Herzkatheteruntersuchung mit einer Rotationsangiographie der Aortenwurzel durchgeführt. Zur Zeit werden wöchentlich ca. 4-8 Patienten für eine mögliche TAVI-Prozedur in der Klinik für Kardiologie, Angiologie und Pneumologie am UKD gesichtet und gegebenenfalls einer entsprechend weiterführenden Diagnostik zugeführt. Im Jahr werden in Summe durchschnittlich 180-220 TAVI-Prozeduren durchgeführt. Der Nachbeobachtungszeitraum hinsichtlich Mortalität und Hospitalisation erstreckt sich über mindestens 36 Monate, erste Teilergebnisse jedoch nach 2 Jahren zu erwarten sind. Die Datenerfassung für das Register soll zunächst bis 2020 laufen.

Siehe auch

Übersetzungen für In diesem Register sollen von allen Patienten aus den Jahren 2010 - 2020 mit symptomatischer Aortenstenose mit der Indikation zum TAVI, die ihr schriftliches Einverständnis gegeben haben, Daten gesammelt werden. Alle Patienten die auf Grund der klinischen Symptomatik bzw. der Vorbefunde einer TAVI-Therapie zugeführt werden sollen, erhalten entsprechend der Routine eine 3D transösophageale Echokardiographie-, sowie nach Ausschluss von Kontraindikationen eine Herz-CT- und eine MRT-Untersuchung. Zudem wird bei allen Patienten eine Herzkatheteruntersuchung mit einer Rotationsangiographie der Aortenwurzel durchgeführt. Zur Zeit werden wöchentlich ca. 4-8 Patienten für eine mögliche TAVI-Prozedur in der Klinik für Kardiologie, Angiologie und Pneumologie am UKD gesichtet und gegebenenfalls einer entsprechend weiterführenden Diagnostik zugeführt. Im Jahr werden in Summe durchschnittlich 180-220 TAVI-Prozeduren durchgeführt. Der Nachbeobachtungszeitraum hinsichtlich Mortalität und Hospitalisation erstreckt sich über mindestens 36 Monate, erste Teilergebnisse jedoch nach 2 Jahren zu erwarten sind. Die Datenerfassung für das Register soll zunächst bis 2020 laufen.

- Tastatur anzeigen
- Tastatur ausblenden

Deutsch

^1234567890ß´

qwertzuiopü+

asdfghjklöä#

<yxcvbnm,.-

Ctrl + Alt.Ctrl + Alt

**5) Inclusion and exclusion criteria**

*Inclusion criteria for TAVI and registry:*

Patients to be included in the registry have to meet the following criteria:

• Symptomatic, severe aortic stenosis

• Decision of the Heart Team for a TAVI procedure

• Willingness and ability to participate in the registry

• Understanding of the informed consent

• Written consent to participate in the study

*Exclusion criteria for the registry:*

• Lack of willingness and ability to participate in the registry

• Lack of written consent

*Exclusion criteria for TAVI:*

• Severe renal impairment (glomerular filtration rate <30 ml / min)

*Exclusion criteria for the cardiac MRI:*

• Presence of MR contraindications (standard MR contraindications):

- Electrical implants such as pacemakers
- Ferromagnetic implants such as aneurysm clips, surgical clips a.m.
- Claustrophobia

**6) Termination criteria**

Termination criteria are an occurrence of the exclusion criteria listed under point 5 (contraindications for MRI, drug intolerance, acute or end stage renal disease).
The patient has the right to revoke his consent to the registry at any time without notice for any reason and without resulting detriment in his treatment.

**7) Possible side effects of medication**

There is no additional medication administered to study patients.
The contrast agents used in the clinical routine for CT and cardiac catheterization (Imeron -> CT; Accupaque-> HK; Bracco, Milan, Italy) are low-osmolar iodinated ionic contrast media (CM). The risk of contrast-induced nephropathy is given with 2%. Appropriate preparation of the patient, contrast-saving examination process and an extended follow-up period can reduce the risk and the occurrence of irreversible kidney damage. Other side effects are nausea and allergic reactions up to anaphylaxis, but that is very rare and with early treatment does not leave any permanent damage. Potentially there is also the risk of contrast agent-induced hyperthyroidism. To minimize that risk, appropriate premedication is performed in patients with pathological thyroid function.

For this study, application of MRI contrast agent is not required. In principle gadolinium contrast agents (Dotarem, Guerbet GmbH, Germany) is used for MRI examinations. In patients with severe renal impairment (GFR <30 ml/min) systemic fibrosis can occur after administration of gadolinium contrats agent. There have been no lasting side effects observed in patients with only mild renal impairment.

The sedative midazolam with a half-life of 10-15 minutes (used for TEE) is very well tolerated and has low respiratory depressive effect. On rare occasions paradoxical drug reactions (with psychic passage syndrome) occur but they are usually short-lasting and without persistent long-term consequences.

**8) Procedure-related complications**

All tests in this study are standard test methods in routine clinical practice. Patients are informed about the risks and potential complications and give their written consent.
Cardiac CT is a low-risk method of investigation, which acquires high-resolution cross-sectional images of the heart with low radiation. The obligatory administration of ionic contrast agent can be associated with adverse effects (see section 7, 9). Cardiac MRI is a routine examination as well. The magnetic resonance imaging uses no radiation and adverse reactions are not described to our knowledge. The recommended standards for magnetic fields are not exceeded and patients with a contraindication to MRI examination (defibrillator etc.), are not included in the registry. Cardiac catheterization too is a routine examination. Complications arise in less than one percent of all cardiac catheterizations. These complications include cardiac arrhythmias, heart attack, embolism, thrombosis, hypersensitivity reactions, infections, vascular injury and bleeding or bruise after puncture, as well as injuries of skin, soft tissue and nerves.

For the recording of routine laboratory parameters no additional blood sampling is required. Among the usual risks is the formation of hematomas in the puncture site. There is a very low risk of local or general infection. In extremely rare cases, there may be a violation of skin nerve with possibly permanent damage.

**9) Risk-benefit analysis**

Since all tests are routinely used for the diagnosis of aortic stenosis and determination of the treatment path, there are no additional risks for patients listed in the registry.

**Risk-benefit analysis of the standard investigation procedure:**

The echocardiographic examinations are not associated with any side effects. The sedation for TEE examination is well tolerated and side effects are rare. The cardiac MRI is an imaging method without ionizing radiation. In compliance with the contraindications (ferromagnetic implants) there is no significant risk. The cardiac CT exam (with modern ECG-gated triggering) is low in radiation dose (8-12 mSv), but the use of contrast agents is obligatory with the associated potential side effects. The cardiac catheterization also bears a comparatively low radiation dose of 3-4 mSv. The risk of acute renal failure can be minimized by appropriate preparation and follow-up. However, it can not be excluded entirely. The risk is about 2%. A routine follow-up period of four years is set.

**10) References:**

1. Makkar RR, Fontana GP, Jilaihawi H, Kapadia S, Pichard AD, Douglas PS, et al. Transcatheter aortic-valve replacement for inoperable severe aortic stenosis. The New England journal of medicine 2012;366:1696-1704.
2. Kodali SK, Williams MR, Smith CR, Svensson LG, Webb JG, Makkar RR, et al. Two-year outcomes after transcatheter or surgical aortic-valve replacement. The New England journal of medicine 2012;366:1686-1695.
3. Messika-Zeitoun D, Serfaty JM, Brochet E, Ducrocq G, Lepage L, Detaint D, et al. Multimodal assessment of the aortic annulus diameter: implications for transcatheter aortic valve implantation. Journal of the American College of Cardiology 2010;55:186-194.
4. Ng AC, Delgado V, van der Kley F, Shanks M, van de Veire NR, Bertini M, et al. Comparison of aortic root dimensions and geometries before and after transcatheter aortic valve implantation by 2- and 3-dimensional transesophageal echocardiography and multislice computed tomography. Circulation Cardiovascular imaging 2010;3:94-102.
5. Jilaihawi H, Kashif M, Fontana G, Furugen A, Shiota T, Friede G, et al. Cross-sectional computed tomographic assessment improves accuracy of aortic annular sizing for transcatheter aortic valve replacement and reduces the incidence of paravalvular aortic regurgitation. Journal of the American College of Cardiology 2012;59:1275-1286.
6. Tamburino C, Capodanno D, Ramondo A, Petronio AS, Ettori F, Santoro G, et al. Incidence and predictors of early and late mortality after transcatheter aortic valve implantation in 663 patients with severe aortic stenosis. Circulation 2011;123:299-308
