# Supplementary material for: Comparison of Manual and Automated Preprocedural Segmentation Tools to Predict the Annulus Plane Angulation and C-Arm Positioning for Transcatheter Aortic Valve Replacement
Source: PLoS One. 2016 Apr 13;11(4):e0151918. doi: 10.1371/journal.pone.0151918 (PMC4830561; doi:10.1371/journal.pone.0151918)
Supplement: S1 Table — (DOC) [file pone.0151918.s004.doc]

**S1 Table. Patient Clinical and Functional Characteristics**

| **Clinical Data** | **Cohort A (n=105)** | **Cohort B (n=20)** | **p-value** |
| --- | --- | --- | --- |
| Age, years ± SD | 80.3±6.4 | 81.8±4.8 | 0.2309 |
| Male, n (%) | 55 (52) | 8 (40) | 0.0007 |
| Weight, kg ± SD | 78.5±17.9 | 71.95±13.7 | 0.1244 |
| Height, cm ± SD | 167.0±13.1 | 166.2±7.9 | 0.8058 |
| Syncope, n (%) | 23 (22) | 4 (20) | 0.8510 |
| CAD, n (%) | 81 (77) | 18 (90) | 0.1972 |
| Previous myocardial infarction, n (%) | 10 (10) | 3 (15) | 0.4462 |
| Previous PCI, n(%) | 46 (44) | 10 (50) | 0.6133 |
| Previous CABG, n (%) | 23 (22) | 4 (20) | 0.8510 |
| Previous valve, n (%) | 3 (3) | 0 (0) | 0.4482 |
| Previous operation on aorta, n (%) | 5 (5) | 0 (0) | 0.3232 |
| Porcelain aorta, n (%) | 15 (14) | 1 (5) | 0.2582 |
| Previous valvuloplasty, n (%) | 2 (2) | 1 (5) | 0.4112 |
| Previous stroke, n (%) | 13 (12) | 0 (0) | 0.0979 |
| Diabetes mellitus, n (%) | 32 (30) | 2 (10) | 0.0600 |
| Arterial Hypertension, n (%) | 103 (98) | 20 (100) | 0.5376 |
| Pulmonary Hypertension, n (%) | 71 (68) | 15 (75) | 0.5177 |
| PVD, n (%) | 23 (22) | 4 (20) | 0.8510 |
| CVD, n (%) | 15 (14) | 3 (15) | 0.9342 |
| COPD, n (%) | 23 (22) | 5 (25) | 0.7632 |
| Atrial fibrillation, n (%) | 26 (25) | 5 (25) | 0.9822 |
| Permanent pacemaker, n (%) | 13 (12) | 4 (20) | 0.3663 |
| Chronic kidney disease, n (%) | 56 (53) | 9 (45) | 0.4981 |
|  | | | |
| **Functional Data** | **Cohort A (n=105)** | **Cohort B (n=20)** | **p-value** |
| Aortic valve area, cm² ± SD | 0.7 ± 0.2 | 0.7 ± 0.2 | 1.0000 |
| Bicuspidie, n (%) | 0 (0) | 0 (0) | 1.0000 |
| MR ≥ grade II, n (%) | 19 (18) | 3 (15) | 0.7415 |
| LVEF <30%, n (%) | 5 (5) | 0 (0) | 0.3232 |
| LVEF 30-44%, n (%) | 15 (14) | 2 (10) | 0.6188 |
| LVEF 45-55%, n (%) | 15 (14) | 4 (20) | 0.5180 |
| LVEF >55%, n (%) | 69 (66) | 14 (70) | 0.7127 |
| Log. EuroSCORE, % ± SD | 21.7 ± 15.8 | 24.5 ± 15.1 | 0.4682 |
| EuroSCORE II, % ± SD | 6.3 ± 5.9 | 5.1 ± 3.3 | 0.3753 |
| STS-PROM-Score, % ± SD | 6.0 ± 4.5 | 5.6 ± 4.0 | 0.7017 |
| NYHA III and IV, n (%) | 73 (70) | 17 (85) | 0.1603 |
| CCS III and IV, n (%) | 20 (19) | 2 (10) | 0.3341 |

Values are mean ± SD or n (%)

CAD=Coronary artery Disease; PCI=Percutaneous coronary intervention; CABG=Coronary artery bypass grafting; PVD=Peripheral vascular Disease; CVD=Cerebrovascular Disease; COPD=Chronic obstructive pulmonary disease; GFR=Glomerular filtration rate; MR=Mitral regurgitation; LVEF=Left ventricular ejection fraction, Calculated either from TEE, TTE or catheter-based; Bicuspidie=Bicuspid valve morphology; Log. EuroSCORE=Logistic EuroSCORE; STS-PROM=Society of Thoracic Surgeons Predicted Risk of Mortality; NYHA=New York Heart Foundation; CCS=Canadian Classification Society.
