# Supplementary material for: Comparison of Manual and Automated Preprocedural Segmentation Tools to Predict the Annulus Plane Angulation and C-Arm Positioning for Transcatheter Aortic Valve Replacement
Source: PLoS One. 2016 Apr 13;11(4):e0151918. doi: 10.1371/journal.pone.0151918 (PMC4830561; doi:10.1371/journal.pone.0151918)
Supplement: S2 Table — (DOC) [file pone.0151918.s005.doc]

**S2 Table. Correspondence in the MSCT-derived Prediction of the PPL** and CAA

| **A-MSCT -derived Prediction of PPL vs. CAA (Cohort B, clinical evaluation)** | | | | | | |
| --- | --- | --- | --- | --- | --- | --- |
| **Deviation of Angulation (n=20)** | **Mean difference** | **Limits** | **95%-CI** | **Pearson (r)** | **R^2^** | **p-value** |
| *LAO/RAO (°) | 0.65±3.39 | -6.00-7.30 | 0.84-0.97 | 0.94 | 0.88 | p<0.0001 |
| *CRAN/CAUD (°) | 4.15±7.48 | -10.52-18.82 | 0.13-0.79 | 0.54 | 0.29 | p=0.0135 |

Values are mean ± SD

Results of the Bland–Altman plots and linear regression analyses for Cohort B comparing the several modalities in LAO/RAO- and CRAN/CAUD-direction.

Limits=Limits of agreement; M-MSCT=Manual derived CAA by MSCT; A-MSCT=Automated derived CAA by MSCT; CAA=Intraprocedural C-arm angulation; LAO and cranial angulation (CRAN) is meant to be positive **(+)**, RAO and caudal (CAUD) direction is signed to be negative **(-)**. LAO=left anterior obliquer; RAO=right anterior oblique; CAUD=caudal; CRAN=cranial; 95%-CI=95% confidence interval.
